# Pollutant particles enhance house dust mite induced type 2 inflammation and the recruitment of monocyte derived Cd11c^+^ Gpnmb^+^ macrophages to the airway lumen

**DOI:** 10.1186/s12989-026-00675-8

**Published:** 2026-04-11

**Authors:** Kirsty Meldrum, Ayokulehin Muse Kosoko, Martin Oliver Leonard

**Affiliations:** 1 Toxicology Department, UK Health Security Agency, Harwell Science and Innovation Campus, OX11 0RQ Didcot, UK; 2 Present Address: In Vitro Toxicology Group, Swansea University Medical School, Swansea University, Swansea, SA2 8PP Wales, UK

**Keywords:** Asthma, Particle pollution, Dust mite, Allergen, Luminal airway, Macrophage, Single cell sequencing, Chemokine

## Abstract

**Background:**

Air pollution particles exacerbate allergic asthma and can enhance inflammatory responses to allergen exposure, but the cellular mechanisms involved remain incompletely defined. We examined how diesel exhaust particles (DEP) enhance house-dust-mite (HDM) inflammatory responses within the lung and characterised potential mechanisms that may contribute to enhanced type 2 (T2) inflammatory responses.

**Results:**

In mice subjected to repeated intranasal exposures, DEP alone had modest effects, whereas DEP + HDM markedly increased type-2 inflammatory indicators (Serum IgE; Airway Il13, Il4 & Tslp) and eosinophilia alongside expansion of Th2 cells. Bulk transcriptomics showed far stronger differential expression in luminal airway cells than tissue, with a DEP + HDM-specific signature enriched for mast cells, alternatively activated macrophages (AAM), and B-cells in the lumen. Combined single-cell proteomic and transcriptomic profiling identified an expanded Cd11c⁺, SiglecF⁻, Apoe⁺, Gpnmb⁺ monocyte-derived macrophage subset (RM.Gp2), which showed increased type 2 chemokines Ccl8 and Ccl24 with DEP + HDM compared to HDM alone. Trajectory analysis placed RM.Gp2 downstream of Ccr2⁺ monocyte derived population, and protein/mRNA data supported a Ccl2–Ccr2-dependent influx that enlarges the RM.Gp2 pool. High-content imaging confirmed increased RM.Mo and RM.Gp2 numbers and higher total luminal Ccl8/Ccl24. F4/80⁺ luminal airway macrophages isolated from DEP pre-treated mice, demonstrated enhanced upregulation of Ccl8 and Ccl24 mRNA in response to ex vivo Il-4/Il-13 treatment, compared to macrophages isolated from control mice. Examination of an additional particle type (CeO_2_ Nanoparticles) in the same exposure model, revealed a shared luminal transcriptomic response and AAM/chemokine programme as with DEP.

**Conclusions:**

Our data suggests that pollutant particles such as DEP may contribute to enhanced HDM induced type 2 inflammation by expanding Ccr2-dependent monocyte-derived macrophages into the airway lumen and licensing a Th2-cytokine-responsive chemokine programme (Ccl8- Ccr8 to recruit Th2 cells; Ccl24-Ccr3 to recruit eosinophils). These findings identify luminal recruited macrophages as important targets in allergic inflammation within the lung, providing insight into potential mechanisms from which exposure and disease mitigation strategies may be developed.

**Supplementary Information:**

The online version contains supplementary material available at 10.1186/s12989-026-00675-8.

## Introduction

Allergic asthma is a potentially life threatening condition with exacerbations commonly triggered by environmental exposures such as allergens, respiratory infections, and air pollution [1]. Co-exposure to environmental triggers is an important consideration for understanding how allergic airway disease develops and how inflammatory exacerbations and sensitisation may be made worse. For example, it has been observed that exposure to traffic associated pollutant DEP can augment allergic T2 inflammatory responses to allergens in humans [2, 3]. From an exposure perspective, humans spend the majority of their time indoors and in urban highly traffic polluted areas, levels of traffic derived particulates can be up to 80% of ambient outdoor levels [4–7]. House dust mite allergens are the most common allergens humans are sensitised to [8, 9] and is also predominantly an indoor allergen [10]. Understanding how these adverse inhalation exposures interact in scenarios of high co-exposure such as indoors in urban centres, remain to be fully understood, especially how patterns of inflammation associated with allergic responses and asthmatic disease may be impacted.

Aeroallergens such as house dust mite disrupt the airway epithelium and trigger the release of alarmins such as TSLP and IL33 as well as chemokines including CXCL5, CCL20 and CCL2 [11]. These signals recruit and activate innate immune cells such as neutrophils, eosinophils, dendritic cells (DCs), and monocytes in the airway mucosa. Antigen-bearing DCs migrate to lymph nodes and, in the presence of type 2 (T2) polarizing cytokines like IL-4, prime naïve T cells to differentiate into GATA3^+^ T_H_2 effector cells. T_H_2 cells produce cytokines (IL-4, IL-5, IL-13) that induce B-cell class switching to allergen-specific IgE and act on local airway mucosa to create a T2 hyperresponsive inflammatory environment in the lungs [11, 12]. Repeated cycles of allergen-induced inflammation lead to progressive airway remodelling and increased risk of severe exacerbations. Although DCs are important in activating the adaptive allergic response, other mononuclear phagocytes (MNPs) also critically modulate allergic inflammation. For example, resident alveolar macrophages (AMs) can suppress early allergic responses, whereas recruited monocytes and their progeny promote allergic inflammation (as shown in murine HDM models) [13]. In mice, Ly6C^+^ monocyte-derived macrophages are required to maintain IL-13–driven lung inflammation and support the homing of T_H_2 cells; depleting these cells reduces HDM-induced airway inflammation [14]. Similarly, immature AMs derived Ccl2, and Ccr2^+^ inflammatory Cd11c^+^ macrophage derived Ccl8, together help recruit T_H_2 and type / group 2 innate lymphoid cells (ILC2) during HDM induced allergic lung inflammation [15]. Additionally, monocytes have been observed to contribute to eosinophil recruitment via production of CCL24, a ligand for the eosinophil receptor CCR3 [16]. Thus, multiple innate immune cell types, beyond classical DCs coordinate to shape the HDM induced allergic T2 immune milieu in the lung.

The particulate component of air pollution has long been examined for its impact on asthma due to its ubiquitous nature and strong association with health effects from inhalation exposure. In particular, the ultrafine fraction of combustion-derived particles (e.g., DEP) is associated with increased asthma morbidity and experimentally to have an adjuvant effect on allergen sensitisation [17]. Experimental approaches have previously observed that DEP alone can have minimal effect on lung inflammation, when administered alone but when given in combination with allergen, exacerbates allergen induced type 2 and type 17 inflammation [18, 19]. An understanding of the mechanisms involved in these enhanced effects, has been suggested to involve recruitment of immune cells, which act to amplify allergen responsiveness. For example, inhalation of diesel exhaust before allergen challenge enhances lower-airway allergic inflammation, as evidenced by increased bronchoalveolar lavage (BAL) eosinophils and T2 cytokines [2]. Interestingly, in this same study diesel pollutant exposure increased Ccl2 expression suggesting that some of DEP’s enhanced effects may be mediated through recruitment of Ccr2 expressing cells including classical monocytes [2]. A functional role for this pathway was recently suggested through observations that blocking monocyte recruitment via the CCR2 chemokine receptor reduced DEP’s enhanced inflammatory effects in a mouse model of allergen exposure [20]. Despite these advances, significant uncertainty still remains regarding the key cellular and molecular mechanisms involved in DEP enhanced allergen induced inflammation, including the relative contribution cellular responses have within different lung compartments.

To address this knowledge gap, we used an established murine model of HDM exposure, which produces a robust type 2 inflammatory allergic response within the lung on repeat administration [21, 22]. Through the application of single cell proteomic (Abseq), single cell and bulk transcriptomic approaches we comprehensively characterise the immunological response underpinning the DEP modifying effects on HDM induced allergic airway inflammation. We focussed on the luminal airway region as a prominent and underexplored site of immunological activity within the lung. Our analysis proposes a cascade of cellular recruitment driven by enhanced macrophage-derived chemokine production, with DEP exposure expanding the pool of monocytes and a sub-type of monocyte-derived recruited Cd11c^+^ Gpnmb^+^ macrophages (RM.Gp2) in the airway lumen. This expansion was associated with amplified type 2 inflammation which we suggest occurs through increased chemokine-driven immune cell recruitment. These findings highlight the critical role of monocyte-lineage cells may have at the site of particulate pollutant exposure and provide a rationale for targeting these cells to mitigate air pollution’s impact on allergic airway disease.

## Methods

### Animal procedures and bronchoalveolar lavage

**Fig. 1:**
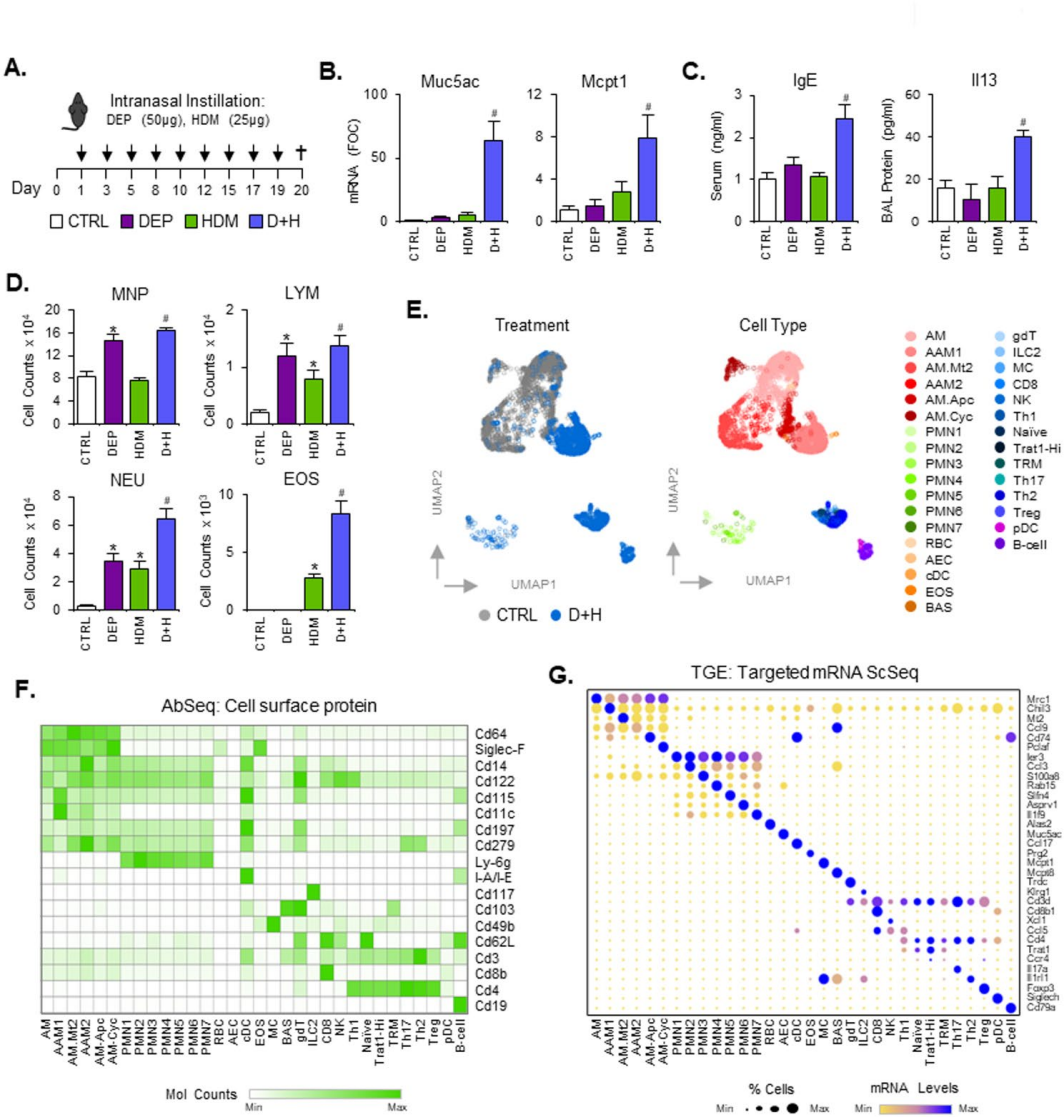
Pollutant particles promote HDM induced type 2 inflammation. Mice (n = 5–7 per group) were instilled with HDM (1.25 mg protein/kg) alone or in combination with DEP (2.5 mg/kg) in a series of 9 repeat exposures over 3 weeks with collection of cells, tissues and blood on day 20 (**A**). Lung tissue was examined for Muc5ac and Mcpt1 levels by RT-PCR (**B**), while plasma levels of IgE and BAL levels of Il13 protein detected using ELISA (**C**). Histochemical changes in LAC profiles were also examined using Wright-Giemsa staining (**D**). *MNP* Mononuclear phagocyte, *LYM* Lymphocyte, *NEU* Neutrophil, *EOS* Eosinophil (**D**). LAC CTRL and D + H groups (pooled n = 3 per treatment) were also examined for single cell expression of inflammatory markers using combined TGE (mRNA) and Abseq (Cell surface protein) (**E–G**) (AMP1 Dataset). Data was visualised as a UMAP with cell types co-localised in condensed multidimensional 2D space (**E**). Cell types were identified based on specific expression patterns and summarised as relative differences in protein levels in a heatmap (**F**) or bubble plot for mRNA (**G**). Results are expressed as Mean +/- SEM for each treatment group. Statistical comparisons of treatments are indicated as * (p < 0.05) when compared to CTRL levels and as # (p < 0.05), when compared to HDM

DEP were sourced from the US National Institute of Standards and Testing (Gaithersburg, Maryland, USA) (Cat# SRM2975). Particles were prepared in PBS with or without HDM (Greer Laboratories, Lenoir, cat# XPB82D3A25, Lot# 327874). Particle preparations were dispersed by sonication (QSonica Sonicators, CT, USA) with 4.2 × 10^5^ kJ/m^3^ immediately prior to lung exposure. Female BALB/c mice between 6 and 8 weeks (Envigo, UK) were anaesthetised with 5% isoflurane in oxygen using a precision vaporizer and intranasally instilled with 25 µl/animal suspensions of either DEP (2 mg/mL), HDM (1 mg/mL) or a combination of both (D + H). Repeated exposure protocol over 3 weeks is as described in Fig. 1A. A 25 µl volume of PBS was used for control (CTRL) exposures. After exposure animals were euthanized using 0.1mL sodium pentobarbital (200 µg/mL) by intraperitoneal injection and exsanguination by cardiac puncture. Plasma was separated from collected blood (EDTA coated tubes) by centrifugation at 1500 xg for 15 min at 4 °C. Bronchoalveolar lavage (BAL) was performed after tracheal catheterisation using three lavages of 500 µl ice-cold sterile PBS. Lavage material was centrifuged at 1500 xg for 10 min at 4 °C and the supernatant from the first lavage was collected for subsequent protein analysis. Lung tissue was subsequently removed, washed in sterile PBS and if not processed immediately was snap frozen for later analysis. BAL cells were counted using a haemocytometer, adjusted to a concentration of 1 × 10^6^ cells/mL and centrifuged using EZ single cytofunnels (ThermoFisher Scientific, UK, cat# 97200125) onto Superfrost™ Plus slides (Menzeil-Gläser Superfrost Plus, ThermoFisher Scientific, UK, cat# 10149870) using a cytospin centrifuge. Differential immune cell visualisation was carried our using a Wright-Giemsa staining (Kwik Diff, ThermoFisher Scientific, UK, cat# 9990700) protocol before mounting and quantification of macrophage (MAC), lymphocyte (LYM), neutrophil (NEU) and eosinophil (EOS) cell numbers. Cells were counted for each animal over three different slides (~ 300 cells/slide). All procedures comply with ARRIVE 2.0 essential guidelines and were performed in accordance with the Animals (Scientific Procedures) Act 1986 (under Project licence number P21FEA4BB), which complies with EU Directive 2010/63/EU. All experimental procedures were reviewed and approved by the local Animal Welfare and Ethical Review Body.

### mRNA isolation and RT-PCR

Lung tissue (post BAL procedure) was homogenised in RLT buffer using MagNA lyser bead disruption (Roche Diagnostics, West Sussex, UK) prior to RNeasy spin column-based RNA isolation. BAL immune cells were directly lysed in RLT buffer and total RNA isolated also using the RNeasy mini kit (Qiagen, Valencia, CA) according to the manufacturers protocol. RNA quantity and purity were determined using nanodrop (ThermoFisher Scientific, UK) and cDNA synthesised using a random hexamer-based protocol (Cat# EP0741, ThermoFisher Scientific, UK). SYBR green based quantitative real time PCR (40 cycles) was carried out using the QuantStudio™ 6 Flex PCR System. Gene specific primers were obtained from the published literature or designed using Primer 3 software and supplied by Integrated DNA technologies, UK (Supplementary Table S1). Fold change in mRNA expression was assessed using the delta-delta Ct method with normalization to the housekeeping gene Hprt1.

### ELISA

Plasma IgE concentrations were quantified using the BD OptEIA ELISA kit (cat# 555248; BD Bioscience, Oxford, UK) according to the manufacturer’s instructions. Cleared BAL fluid protein levels for Il13 (Cat# DY413), Il4 (Cat# DY404), Il5 (Cat# DY405), Il33 (Cat# DY3626), Tslp (Cat# DY555), Csf2 (Cat# DY415), Il1α (Cat# DY400), Ccl11 (Cat# DY420), Ccl17 (Cat# DY529), Ccl20 (Cat# DY760) and Ccl2 (Cat# DY479) using DuoSet ELISA kits from Bio-Techne (Abingdon, UK). Samples were analyzed in duplicate. Absorbance was measured at 450 nm with background levels at 570 nm using a plate reader (Bio-Tek Synergy HT). Extrapolation of protein levels was carried out from a standard curve of recombinant proteins.

### Single cell multi-omic analysis: targeted gene expression

Bronchoalveolar lavage cells were processed for multi-omic single cell analysis using a combination of antibody based immune cell surface marker protein detection (Abseq) and targeted gene expression (TGE) profiling using the Rhapsody express system and scanner (BD Biosciences, UK). 18 Abseq antibodies covering a range of myeloid and lymphoid immune cell types was used for profiling (Supplementary Table S3). A combination of 397 genes from the mouse immune cell panel (Cat# 633753, BD Biosciences UK) (Supplementary Table S4) and 93 genes from a custom designed panel (Supplementary Table S5) was used for TGE profiling. Multiplexing of samples was carried out using Sample-tag antibodies (Cat# 633793). Procedures were carried out according to the manufacturer’s instructions with ~ 2 × 10^5^ BAL cells starting material for each sample. Briefly, non-specific Fc Receptor sites were blocked with Mouse BD Fc Block™ prior to Sample tag and Abseq antibody labelling for 20 min at room temperature. After washing and equal proportion sample pooling, cells were stained with viability markers Calcein-AM and DRAQ7 and counted using the Rhapsody scanner. Cells were passed through a 37 μm cell filter and between 5000 and 10,000 viable cells were loaded onto each cartridge. After cell lysis and bead capture of mRNA and antibody oligonucleotide tags, on bead cDNA synthesis was carried out according to manufacturer’s guidelines (Cat# 633773). Purification of cDNA, Abseq and Sample-tag PCR products was carried out using AMPure XP magnetic beads (Beckman Coulter, cat#A63881). Index library preparation was performed with 2.5ng/µL TGE cDNA and 1ng/µL for Abseq and Sample-tag PCR products using a further 6 PCR cycles of amplification. cDNA was quantified using Qubit and Agilent Tapestation and pooled (~ 85/17/2% mRNA/AbSeq/Sample-tag ratio) to achieve a final concentration of 5 nM. Libraries were spiked with 15% PhiX to increase sequence complexity and sequenced (100PE) on a HiSeq X-Ten sequencer (Illumina). FastQ output files were annotated to reference libraries to identify cell label and unique molecular identifier (UMI) sequences using the BD Rhapsody™ Analysis Pipeline Version 1.2 (BD Biosciences, UK). The number of distribution-based error correction (DBEC) molecules (mols) per cell was then extracted, multiplets and undetermined Sample Tag cells removed. Quality control, removal of low quality cells and low expressed genes, as well as normalisation (10,000 mols per cell) was carried out using SeqGeq version 1.7.0 (FlowJo LLC, US) software. Immune cell populations were identified using a combination of manual annotation from cell type marker expression and Seurat 3.0 plugin based clustering and visualised using UMAP dimensionality reduction of highly diverse expressed genes. Differential expression between cell populations was calculated and expressed as fold change v FDR q-value significance. Two separate datasets for TGE+Abseq were generated (AMP1 and AMP2). AMP1 comprised two matched samples, CTRL and D + H generated by pooling BAL cells from three separate animals per treatment. AMP2 comprised six matched samples, from three separate animals per treatment (CTRL and D + H) each individually tagged. Both datasets are described across Figs. 1 and 4 and Supplementary Figures S2 & S3.

### Single cell multi-omic analysis: whole transcriptome analysis

Bronchoalveolar lavage cells were processed for multi-omic single cell analysis using a combination of antibody based immune cell surface marker protein detection (Abseq) and whole transcriptome analysis (WTA) profiling using the Rhapsody express system and scanner (BD Biosciences, UK). 18 Abseq antibodies identifying a range of myeloid and lymphoid immune cell types were used for profiling (Supplementary Table S3). Multiplexing of samples was carried out using Sample-tag antibodies (Cat# 633793, BD Biosciences UK). Procedures were carried out according to the manufacturer’s instructions with ~ 2 × 10^5^ BAL cells as starting material for each sample. Prior to Abseq and Sample tag labelling, non-specific Fc Receptor sites were blocked with Mouse BD Fc Block™. Cells were filtered through a 37 μm cell filter prior to loading 20,000 viable cells onto the microwell capture cartridges. Whole transcriptome profiling was carried out using the BD Rhapsody™ WTA Amplification Kit from BD Biosciences according to the manufacturer’s instructions (Cat# 633801, BD Biosciences UK). Index library preparation was performed with 2 nM WTA RPE PCR products and at a concentration of 1ng/µL for Abseq and Sample-tag PCR products using a further 8 PCR cycles of amplification. cDNA was quantified using Qubit and Agilent Tapestation and pooled (~ 80/18/2% mRNA/AbSeq/Sample-tag ratio) to achieve a final concentration of 5 nM. Libraries were spiked with 1% Phix to increase sequence complexity and sequenced (100PE) on a HiSeq X-Ten sequencer (Illumina). FastQ output files were annotated to reference libraries to identify cell label and UMI sequences using the BD Rhapsody™ Analysis Pipeline Version 2.0 (BD Biosciences, UK). DBEC molecules per cell was then extracted, multiplets and sample tag unlabelled cells removed. Quality control, removal of low quality cells and low expressed genes, as well as normalisation (10,000 mols per cell) was carried out using SeqGeq version 1.7.0 (FlowJo LLC, US) software. The plug-in Lex-BDSMK was used to separate out the different samples based on sample tag ID. Immune cell populations were identified using a combination of manual annotation from cell type marker expression and Seurat 3.0 plugin based clustering and visualised using UMAP dimensionality reduction of highly diverse expressed genes. Trajectory analysis was carried out using the software package monocle3 [23]. The final dataset comprised eight samples of BAL cells, from four separate animals per treatment (HDM and D + H) each individually tagged (Figs. 4 and 6 and Supplementary Figures S4, S5 & S7).

### Bulk seq and cell type interpolation

Total RNA from Lung tissue (TISS) and luminal airway cells (LAC) were analysed for integrity and purity using an Agilent 2100 Bioanalyzer. Samples with an RNA integrity number (RIN) above 7.0 were processed for bulk-seq analysis. cDNA libraries underwent paired end (150PE) sequencing for transcriptomic analysis using the DNBseq platform (BGI Genomics, HK). 20 million clean reads for each sample were trimmed to remove remaining adapters and other variable sequences, followed by gene level annotation using the Hg38 human or Mm39 reference genome builds using CLC Genomics Workbench software (CLCBIO, Aarhus, Denmark). CLC software was also used to perform differential expression using a general linearised model with a negative binomial distribution. For comparative analysis of select genes between treatment groups data was also extracted as reads per kilobase per million (RPKM). Groups of cell type specific genes were used for cell type interpolation of bulk-seq data and were collated through manual curation of the literature, the Immgen database and a lung mouse cell atlas [24–27] (Supplementary Table S6). Relative quantification of cell type levels from bulk-seq was then calculated using an enrichment score based method [28, 29] as the average differential expression score (DES) (Log2(FC) x -Log10(FDR q Value) across genes within each cell type group.

### Immunofluorescence and High content imaging

BAL cells were collected onto cytospin slides for 4 min (min) at 480 rpm and fixed with 4% (v/v) paraformaldehyde in PBS (Sigma) for 10 min. Slides were then washed three times in PBS followed by permeabilization with 0.2% (v/v) Triton-X 100 / PBS for 15 min at room temperature (RT). After a second wash step, slides were incubated with blocking buffer (3% (v/v) Donkey serum, 3% (v/v) FBS in PBS). For indirect immunofluorescent staining of Ccl8 and Ccl24, slides were then incubated with primary antibodies in antibody dilution buffer (0.2% (v/v) Donkey serum, 0.2% (v/v) FBS in PBS) for 2 h (hrs) at RT. After washing three times at 5 min with PBS, slides were incubated with secondary antibodies (Donkey anti rabbit and anti-goat) and wash a final time in PBS (3 × 5 min), mounted with DAPI containing antifade media (Cat# P36931, Thermofisher Scientific, UK) and scanned using high content imaging (ImageExpress PICO, Molecular Devices). For directly conjugated antibody staining (Cd11c, SiglecF, MHCII) slides were incubated in antibody solution directly after the blocking step for 2 h at RT, followed by washing 3 times with PBS (5 min each) before mounting and scanning. Antibody details and dilutions are provided in supplementary table S7. CellReporterXpress software was used to identify and quantify cell type numbers from direct conjugated antibody experiments. For chemokine quantification of protein granule area and intensity, MetaXpress software was used to call positive cells and protein levels / per cell.

### F4/80 Macrophage isolation and ex vivo culture

LAC were isolated using bronchoalveolar lavage (1 Lung per sample) and processed for murine F4/80 positive macrophage cell isolation using an EasySep Positive selection Kit (StemCell Technologies, Grenoble, FR) (Cat# 100–0659) according to manufacturer’s instructions. Lung tissue was also used to isolate F4/80 positive cells using 1 whole lung per samples. Briefly, lung digestion was performed using collagenase/hyaluronidase (Cat# 07912, StemCell) with DNase I (Cat#07900, StemCell) before filtration through a 70 μm cell strainer, ammonium chloride treatment to remove RBCs and cell pellet resuspension in Ca/Mg free PBS containing 2% FBS and 1 mM EDTA. LAC and tissue cells were treated with Mouse BD Fc Block™ for 15 min at room temperature to block nonspecific binding prior to magnetic column separation. F4/80 cells were resuspended in RPMI media containing 0.1%(w/v) human serum albumin and penicillin/streptomycin (100U/mL) and plated at a concentration of 2 × 10^4^ cells/mL onto low attachment 96 well plates (Cat# CLS3474, Merck). Cells were immediately treated with combinations of HDM (Greer Stallergenes) and murine IL-4 and IL-13 (Bio-Techne, UK) for 12 h before mRNA isolation and PCR analysis.

### Statistical analysis

Statistical significance between groups was carried out with Graphpad Prism Software version 9 using one-way ANOVA / Fisher’s LSD Test unless otherwise stated. Results are expressed as mean ± standard error of the mean (SEM).

## Results

### DEP and HDM co-exposure amplify type 2 inflammatory responses in the lung

To investigate the enhanced effects of DEP on dust mite induced inflammatory responses, we exposed 6–8 week old mice to a regimen of 9 repeat treatments over 3 weeks (Fig. 1A). Our focus was to capture active cellular and molecular processes involved in the establishment and maintenance of inflammatory patterns in the lung, and therefore used a short time point of 24 h after the last exposure to perform characterisation analysis. DEP or HDM alone did not result in any change in inflammatory or allergic airway disease markers Muc5ac, Mcpt1 and Il13 (Fig. 1B-C). However, when combined (D + H) there was a statistically significant increase compared to control (Fig. 1B-C). Plasma levels of IgE (Fig. 1C) and BAL protein levels of the type 2 inflammatory cytokines Il4, Tslp and Ccl17 (Supplementary Figure S1A) also followed the same pattern. Attempts to establish a HDM specific IgE assay to assess allergen specific sensitisation were unsuccessful during the project timeframe (Data not shown). BAL cell counts revealed an increase in MNPs with DEP treatment, which was not enhanced by co-treatment with HDM (Fig. 1D) This response pattern was also observed for BAL protein levels of Csf2 and Ccl20 (Supplementary Figure S1A). Lymphocyte and neutrophil counts were increased with DEP and HDM treatments alone and further increased upon co-treatment only for neutrophil numbers (Fig. 1D). Eosinophils were not altered by DEP alone but were increased with HDM treatments and further enhanced by co-treatment exposures (D + H) (Fig. 1D).

### Characterisation of luminal airway immune cell population changes after DEP + HDM co-exposure using protein and mRNA based single cell sequencing

Further examination of luminal airway cell (LAC) populations from BAL was next carried out using a combined method for TGE ScSeq of mRNA levels and Abseq profiling for cell surface protein levels (Fig. 1E-G). Results from the AMP1 dataset are displayed (Fig. 1E-G). CTRL (2415 cells) and D + H (2186 cells) population data are represented as dimensionality reduction UMAP plots, where cells with a similar expression pattern are co-localised in 2-dimensions (Fig. 1E). Cells were analysed using amplicon based detection of 490 inflammatory genes for TGE and 18 cell surface protein markers (Abseq), chosen for a comprehensive characterisation of immune cell populations. Cell types were identified through their unique expression pattern of protein (Fig. 1F) and mRNA (Fig. 1G) markers, curated from previous experimental datasets [30–37]. Within the CTRL population, we identified AMs, metallothionein-2 high expressing macrophages (AM.Mt2), macrophage like antigen presenting cells (AM.Apc) and proliferating/cycling macrophages (AM.Cyc) (Fig. 1E). No other cell type was present within the control population to any significant extent. Cells obtained from the D + H treatment group displayed a large difference in composition compared to CTRL, including a predominant Chil3 + ve alternatively activated macrophage (AAM1) population. Lesser quantities of other macrophage populations identified in CTRL were also present (Fig. 1E). Neutrophils (PMN1-7), eosinophils (EOS), dendritic cells (cDC2 & pDC), mast cells (MC), basophils (BAS), ILC2 cells, NK cells, B cells and different types of T-cells (gδT, CD8, Naïve, T_H_1, Trat1-Hi, TRM, T_H_17, T_H_2, Treg) were identified in the D + H Scseq but not the CTRL population (Fig. 1E) consistent with an active inflammatory environment within the airway lumen in these mice. We confirmed these changes in immune cell populations within the airway lumen through a further set of exposures and assessment by TGE ScSeq analysis (Supplementary Figure S2B: AMP2 dataset). For this additional dataset, categorisation was carried out using TGE expression only, without Abseq protein labelling, and for the most part the same cell types were identified. Two subtypes of PMNs identified in the original AMP1 dataset were not resolved in the AMP2 dataset. With the additional data from AMP2, two further types of T-cells (CD4^+^/CD8^+^ & CD4^ccr7+^) could be identified (Supplementary Figure S2C). There was a high degree of corelation between Scseq AMP1 and AMP2 datasets for D + H induced gene expression (Supplementary Figure S2D). Quantification of cell type numbers altered with D + H treatment compared to CTRL revealed statistically significant decreases in AMs and AM.Apc, while significant increases in the majority of other cell types including subtypes of myeloid, PMN and T-cells (Supplementary Figure S2E). An assessment of mRNA changes due to D + H treatment from the same population of cells used for ScSeq (AMP2 dataset) was also investigated using a bulk-seq method (Supplementary Figure S2F). This demonstrated a high degree of corelation for all markers used for immune cell type identification, except for eosinophil associated genes (e.g. Prg2, Epx and Retnlg). Difficulties in the ability of ScSeq to identify eosinophil transcriptomes has been observed previously due to lower mRNA levels and high levels of RNAse enzymes [38, 39], but changes in our amplicon datasets still indicate a sufficient degree of detection for relative quantification purposes (Supplementary Figure S2F).

### Bulk-seq transcriptomic responses to DEP, HDM and D + H pulmonary exposure

**Fig. 2:**
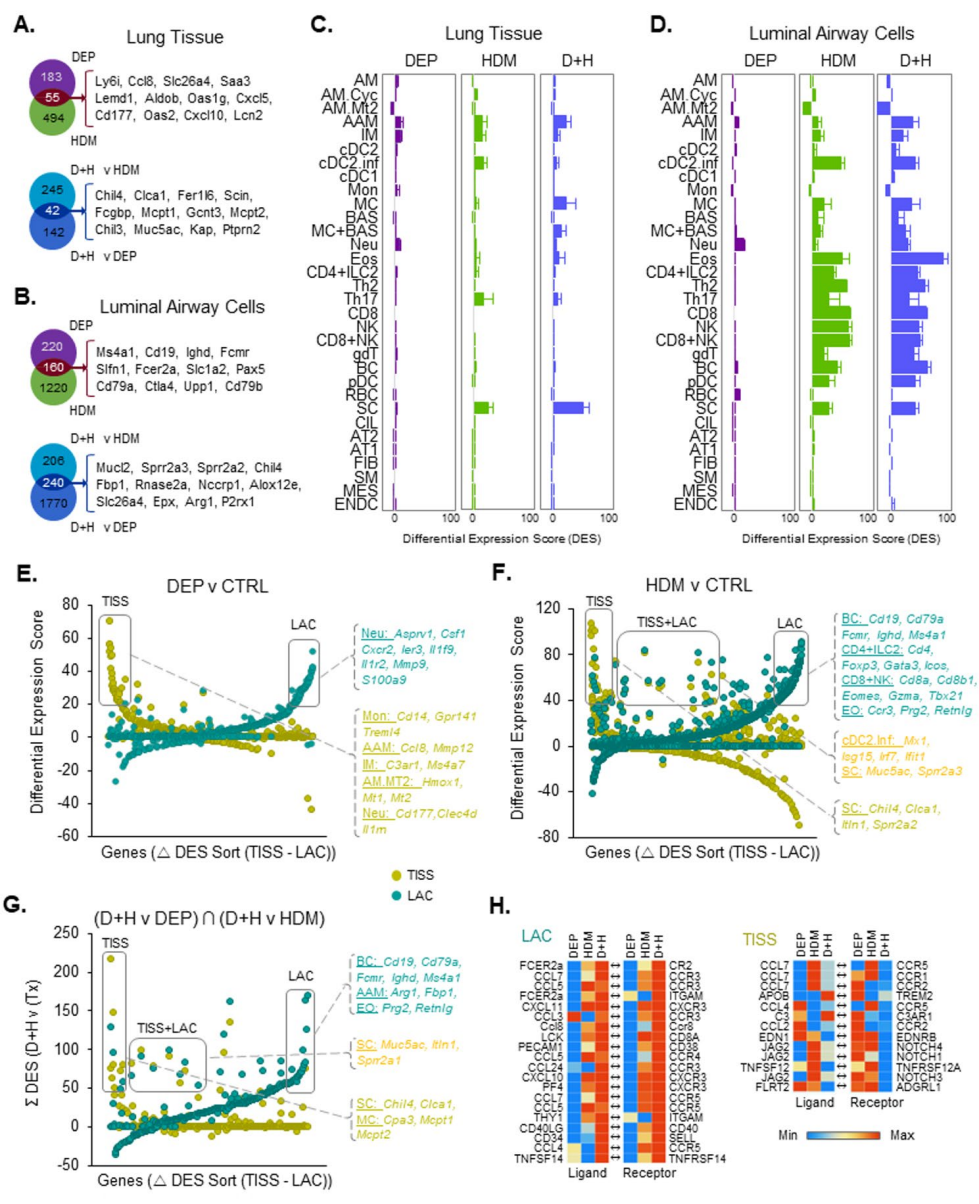
Transcriptional profiling reveals compartment specific responses associated with DEP enhanced HDM effects. Mice (n = 5–7 per group) were treated with HDM and DEP alone or in combination as described in Fig. 1A. LAC and lung tissue were isolated separately and processed for mRNA extraction and bulk-seq whole transcriptome analysis. Differentially expressed genes (> 2 fold change; FDR p value < 0.05) were identified with unique and common genes across comparisons displayed as numbers of genes in Venn diagrams (**A**, **B**). The 12 most highly upregulated genes within Venn diagram intersects are also displayed (**A, B**). Cell interpolation of bulk-seq was used to identify changes in cell types. A differential expression score (DES) for each treatment versus CTRL was calculated for each cell type specific gene and results expressed as mean +/- SEM for genes within a cell type (**C**, **D**). A comparison of LAC and lung tissue (TISS) bulkseq gene expression changes identified in **A** and **B** was made through sorting genes based on their difference in DES scores between compartments (Δ DES Sort) (**E**–**G**). Those genes altered by combined D + H exposure only was identified as the intersect (∩) of combined versus single exposures (**G**). Cell specific genes from cell interpolation categorisation were highlighted within compartment specific and common groupings (**E**–**G**). Ligand receptor pairs from the CellPhoneDB database, whose expression were both differentially expressed by D + H treatment (1.25 FC; FDR p value < 0.05) were identified. (**H**). For the LAC compartment 20 of 170 significant pairs identified are displayed (H; Left panel) while all of the TISS pairs are displayed (H; Right panel). Murine equivalent pairs substituted for human database pairs are displayed as lower case. Heatmap values are normalised DES scores. Cell type abbreviations are indicated in the abbreviatiosn section)

We next used bulk-seq to investigate whole transcriptome changes within the lung in response to repeated DEP and HDM exposure over 3 weeks (Fig. 2). Datasets from both lung lavage luminal airway cells (LAC) and corresponding lung tissue (TISS) were generated. HDM treatment resulted in a substantially greater number of differentially expressed genes than DEP, which was observed for both LAC and TISS samples (Fig. 2A-B, top panels). Combined DEP and HDM exposures (D + H) were then compared to either exposure type alone. Those genes common between both comparisons were identified as uniquely expressed by the combination alone (Fig. 2A-B, lower panels). This revealed 42 and 240 genes for TISS and LAC compartments respectively (Supplementary Tables S9,S10), all with potential mechanistic involvement in enhanced inflammatory effects by DEP. Of further interest was the observation that the number of differentially regulated genes from HDM exposure was substantially higher in LAC samples than TISS, an observation not found with DEP exposures (Fig. 2A-B). Upon further examination of Bulk-seq data and comparison with cell type specific markers identified in the ScSeq datasets, a large proportion of differentially regulated genes were identified as unique cell type specific markers (Fig. 2A, B). We next carried out cell type interpolation of both TISS and LAC datasets to quantify changes in these cells across treatments (Fig. 2C-D). The specificity of interpolation gene sets for cell type was confirmed through comparison to AMP1, AMP2 and published ScSeq lung datasets [24, 25, 30, 35, 40] (Supplementary Figure S3A-B). Macrophages typically present within control lungs (AM, AM.Cyc & AM.Mt2) displayed no change or a decrease in levels (Fig. 2C, D). AAM however, displayed increases with either DEP or HDM treatment, which was further enhanced by the combination D + H, an effect more pronounced in the LAC compartment. Changes in interstitial macrophages (IM) were also observed, with enhanced combinatorial D + H effects observed only within the LAC compartment. Mast cells were also enhanced with D + H when compared to either treatment alone, an effect consistent across both TISS and LAC (Fig. 2C, D). Eosinophils and CD4 + ILC2 interpolated cell quantities were also increased (Fig. 2D), an effect not observed with other CD4, CD8 or NK cell categories. Of the remaining immune cell types examined, gδT cells and B cells only displayed enhanced D + H effects, when compared to single treatments alone, an effect only observed in the LAC compartment. Of the structural cells within the lung, secretory goblet cells alone increased with HDM and further increased with D + H treatment (Fig. 2C, D). The changes observed in these sets of data are consistent with enhanced HDM induced type 2 inflammation upon co-exposure with DEP, where DEP alone has limited or no effect on these specific responses.

### Compartment and treatment specific responses identified through bulk-seq transcriptomic comparison

More broadly, it could be observed that changes caused by DEP were observed to a lesser extent than with HDM, and the differences in cell types were more pronounced in the LAC than TISS compartments (Fig. 2C-D), consistent with the number of significantly differentially expressed genes (DEGs) (Fig. 2A-B). Compartment dependent gene expression changes due to DEP and HDM treatment were examined in more detail by direct compartment comparisons (Fig. 2E-F). DEP induced changes were for the majority of genes exclusive for the TISS and LAC compartments with relatively few genes regulated to the same level between both (Fig. 2E). For HDM, this exclusivity was generally the same between TISS and LAC except for a strong inflammatory cDC2 cohort of increased expression, present at the same level for both compartments (Fig. 2F). While common responses like this may be a true reflection of both compartments’ inflammatory response, we cannot be entirely confident that some luminal airway cells remained in the TISS compartment after lavage as contributors to differential transcriptional signals in the TISS samples. However, given the exclusivity for the majority of transcriptomic responses between TISS and BAL samples, we can be relatively confident that the majority of these signals are a true reflection of tissue versus luminal airway differential response. Gene expression changes also indicated a compartment selective difference in cell types with DEP and HDM treatments. For example, increases in lymphocyte markers from HDM treatment, were exclusively within the LAC (Fig. 2F). To further examine the mechanism through which DEP was exerting its enhanced effects on HDM induced inflammation, we next examined those genes, which were further up or down regulated on D + H co-treatment statistically significant beyond levels from either treatment alone (Fig. 2G). This intersection of genes represents either additive, regressive or synergistic regulation. Genes within this selection, identified mast cells and secretory cells predominantly increased within the tissue compartment, while B-cell and AAM were selectively increased in the LAC compartment (Fig. 2G). Sensitivity analysis was also carried out for differential gene expression across both compartments to investigate limits of read count detection. Differentially regulated genes (FDR < 0.05) for LAC D + H v CTRL, with a CTRL RPKM value greater than 0.01 were selected and represented 95% of DEGs (Supplementary Figure S3C). These same genes were then examined for RPKM expression in TISS CTRL and D + H datasets (Supplementary Figure S3D). 99.6% of genes displayed RPKM values greater than 0.01, indicating a reduced level of responses within the TISS compartment is not due to a limit of detection. There was also no large difference in sample group variance across LAC and TISS datasets that could explain differences between compartments (Supplementary Figure S3E-F). To provide insight into immune cell infiltration dynamics into lung tissue and airway compartments, we next interrogated bulk-seq gene sets for cell surface ligand and receptor partner gene co-expression using the CellPhoneDB database (Fig. 2H). 165 receptor – ligand pairs where both partners were differentially expressed on D + H treatment were identified in the LAC dataset, while 13 were identified for TISS (Supplementary Tables S11, S12). The heatmap in Fig. 2H (left panel) displayed the top 20 LAC pairs, which for the majority displayed enhanced expression with D + H when compared to either DEP or HDM treatment alone. LAC pairs included Ccl8:Ccr8, a key axis for ILC2 and CD4 T_H_2 cell recruitment into the lung after HDM exposure [15], CCL24:CCR3, which is important for eosinophil and mast cell recruitment [16] and Ccl2:Ccr2 which contributes to DEP enhanced allergen induced airway disease through monocyte recruitment [20]. We also examined whether signals derived in the LAC may interact with the TISS compartment to recruit cells. Analysis of significantly differentially expressed ligand - receptor partners across the LAC – TISS compartments respectively, did not identify any directionality consistent with recruitment of cells along this trajectory (Supplementary Figure S8A).

### Cerium dioxide nanoparticles (CEP) and DEP enhance HDM induced type 2 inflammation and share a program of transcriptional alterations in the LAC compartment

**Fig. 3:**
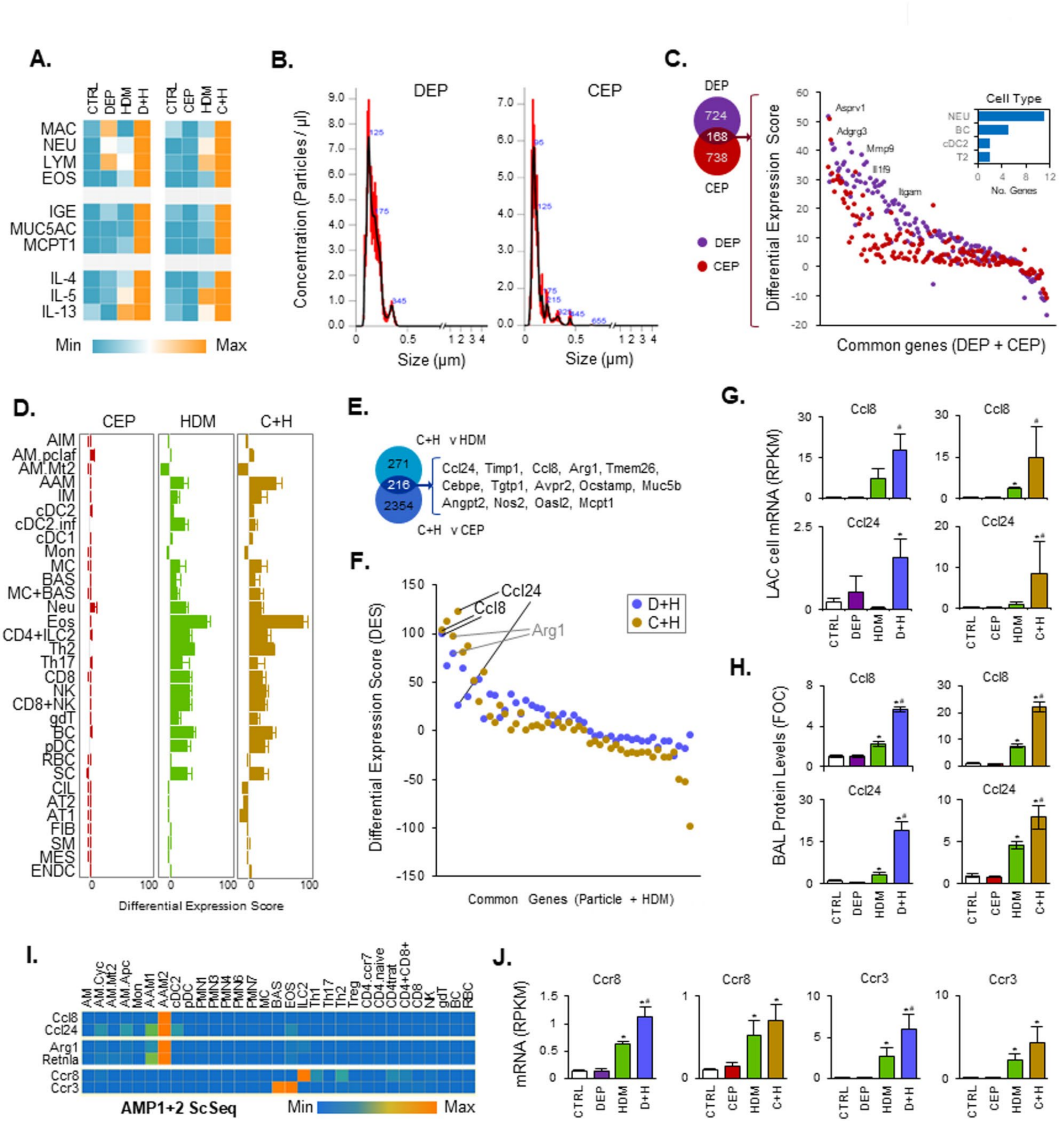
Comparison of DEP and CEP particles for enhanced effects on HDM induced type 2 inflammation. Data was extracted from our previous study [41], examining the adjuvant effect of cerium dioxide nanoparticles (CEP) in the same repeat HDM exposure model and compared to DEP effects in this study. Histochemical staining for different cell types (MAC, NEU, LYM, EOS), Muc5ac and Mcpt1 RT-PCR, plasma IgE and Il4, Il5 and Il13 BAL fluid protein levels were normalised to maximum and displayed as a heatmap for CEP and DEP datasets (**A**). Particle size distribution was examined using nanosight nanoparticle tracking analysis (**B**). A new set of bulk-seq data was generated from CEP LAC mRNA obtained from our previous study (*n* = 5–6 per treatment group) and compared to DEP bulk-seq data (**C**, **D**, **E**, **F**, **G**, **J**). Common genes altered by particle alone treatment (1.5 fold change, FDR < 0.05) are displayed sorted based on DES (**C**) and the numbers of cell type specific genes per cell interpolation cell type are displayed (C; insert panel). Cell interpolation of LAC bulk-seq is displayed for the CEP dataset (**D**) and those highest regulated genes induced by combined CEP + HDM (C + H) exposure are indicated (**E**). Those genes that were identified as common between D + H and C + H datasets (enhanced above both HDM and particle treatments alone) are displayed as DES values (**F**). Ccl8 and Ccl24 RPKM from bulk-seq data are shown (**G**). BAL fluid Ccl8 and Ccl24 protein levels were determined and expressed as fold over control levels (FOC) (**H**). TGE Scseq data from D + H exposures was examined for cell type specific expression of Ccl8, Ccl24, Arg1, Retnla and the chemokine receptors Ccr8 and Ccr3 (**I**). Bulk-seq RPKM levels for chemokine and chemokine receptor expression in LAC are displayed (**J**). Results are expressed as Mean +/- SEM for each treatment group. Statistical comparisons of treatments are indicated as * (*p* < 0.05) when compared to CTRL levels and as # (*p* < 0.05), when compared to HDM

We have previously demonstrated that cerium dioxide nanoparticles (CEP) in the same exposure model display enhanced effects for HDM induced pulmonary type II inflammatory responses, at similar doses to DEP used in this study [41]. Comparison of inflammatory cell and mediator levels for the same endpoints investigated in this study, revealed both particle types induced near identical “particle” + HDM enhanced effects above treatment with either particle or HDM alone (Fig. 3A). With these similarities and the findings that the size distribution of these particles was highly similar (Fig. 3B), we performed bulk-seq analysis on LAC cells from our previous CEP study, to compare to DEP induced changes in order to uncover common mechanisms of immune cell recruitment and enhanced inflammatory effects in the airway lumen. 168 differentially expressed genes common to both particle type exposure was revealed, with similar magnitude of expression (Fig. 3C). Cell interpolation of LAC CEP datasets (Fig. 3D) demonstrated patterns similar to those found with DEP and HDM (Fig. 2D). CEP alone had little effect on cell type changes within the airway lumen, similar to DEP. It did however enhance levels of HDM induced AAM, IM, MC and eosinophils (Fig. 3D) similar to DEP enhanced effects (Fig. 2D). Direct comparison of CEP + HDM enhanced genes identified in Fig. 3E to those enhanced with combined D + H treatments identified AAM genes as the most significantly altered including Arg1, Ccl8 and Ccl24 (Fig. 3F, G). Protein levels for Ccl8 and Ccl24 in BAL fluid were also increased above HDM and particle alone treatments for both C + H and D + H combinations (Fig. 3H). Cell type expression of Ccl8 and Ccl24 was restricted to AAM1 and AAM2 Scseq populations, with AAM2 displaying the highest mRNA expression (Fig. 3I). Receptor partners for these chemokines were expressed predominantly in ILC2 and CD4.Th2 cells for Ccr8, and in basophils and eosinophils for Ccr3 (Fig. 3I). mRNA levels of these chemokines were increased with HDM treatment and enhanced with D + H treatment (Fig. 3J). C + H treatment also increased mRNA expression of Ccr8 and Ccr3 above HDM levels in LAC but this was not statistically significant (Fig. 3J).

### Whole transcriptome single cell seq analysis identifies luminal airway recruited and resident mononuclear phagocyte populations with differential expression of alternatively activated / type 2 inflammatory signals

**Fig. 4:**
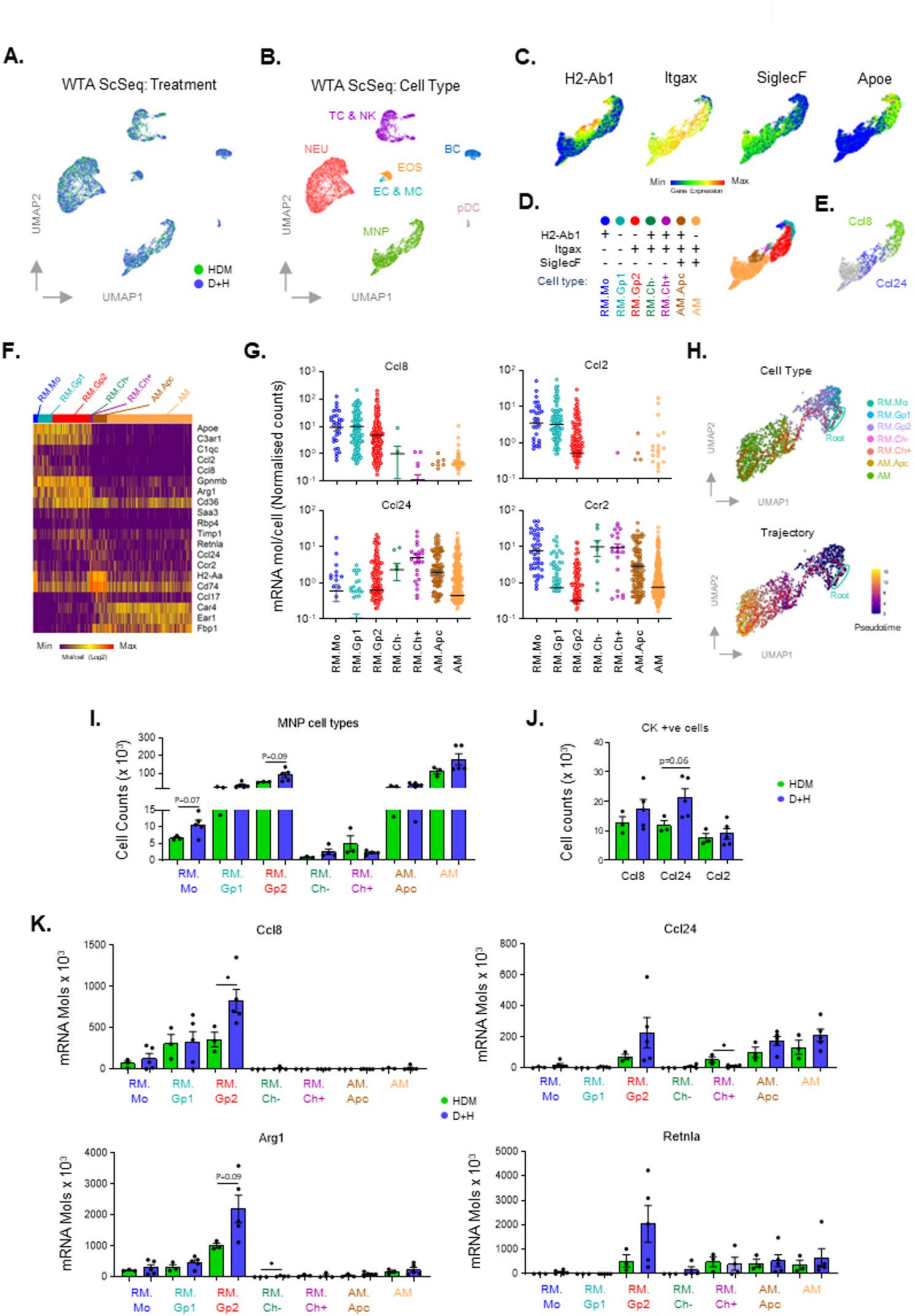
WTA Scseq analysis identifies DEP enhanced MNP cell type specific type 2 inflammatory gene expression. WTA Scseq analysis was carried out on LAC isolated from HDM (*n* = 3) and D + H (*n* = 5) repeat exposed mice (9 times over 3 weeks) and cell types (NEU; Neutrophil, TC; T-cell, NK; Natural Killer cell, BC; B-cell, EOS; Eosinophil, EC; Epithelial cell, MC; Mast cell, MNP; Mononuclear phagocyte, pDC; Plasmacytoid dendritic cell) identified and visualised using UMAP plots (**A**, **B**). MNP marker expression on MNPs is displayed as UMAP (**C**) and sub-populations identified (**D**). Cells positive for Ccl8 and Ccl24 are displayed (**E**). Expression of MNP RM and AM markers for each cell within these sub-populations is shown, normalised to the maximum molecule counts per cell for each gene (**F**). mRNA molecule counts per cell was normalised to total cell transcriptome molecule counts and levels for Ccl8, Ccl24, Ccl2 and Ccr2 across MNP sub-populations displayed for each cell (**G**). Zero count cells were not included. Trajectory analysis of MNP sub-populations was carried out using Monocle3 and RM.Mo cells as the root population, with cell types (**H**; Upper panel) and pseudotime progression (**H**; Lower panel) displayed. Total numbers of MNP cell types per lung are displayed (**I**). Cells positive (+ ve cells) for chemokine expression across all LACs are displayed as total cells per lung (**J**). Total mRNA molecules per lung LAC for chemokine and AAM genes are indicated for each MNP sub-population (**K**). HDM treatments are indicated as green and D + H as blue. Results are expressed as Mean +/- SEM for each treatment group. Statistical comparisons of D + H v HDM were carried out using unpaired student t-test with significance indicated as * (*p* < 0.05) or as specific p values

We next examined how DEP modifies HDM induced LAC immune responses using whole transcriptome ScSeq analysis of HDM and D + H exposures (Fig. 4A), a technique that analyses all gene expression changes as opposed to targeted gene analysis. Granulocyte, mononuclear phagocyte (MNP) and lymphoid cell populations were identified (Fig. 4B; Supplementary Figure S4A). Cell surface protein and mRNA expression for MHCII (H2-Ab1), Cd11c (Itgax), SiglecF and Apoe identified the main sub-populations of MNP cells (Fig. 4C, Supplementary Figure S5B). Two broad categories were found; recruited macrophages (RM) identified as positive for Apoe and Itgam expression, and resident AM identified as positive for SiglecF expression [30, 42] (Fig. 4C; Supplementary Figure S4A). Sub-populations of MNPs were further classified based on unique patterns of gene and protein expression (Fig. 4C, D,F; Supplementary Figure S5A, Supplementary File 2). RM.Mo were positive for H2-Ab1 and had high levels of Ccr2 expression typical of classical monocytes. Two recruited macrophage populations had high levels of Gpnmb (RM.Gp1 and RM.Gp2). These cells have recently been identified as a type of monocyte derived macrophage (MoDM) recruited to the lung during injury and fibrosis [43–46]. These studies identified the cells as uniquely expressing Spp1, Fabp5, Trem2, Ccl7, Ccl2, Ctsl and Cd63, distinguishing them from other immune cell types. In our study, these markers were differentially expressed across the two RM.Gp sub-populations (Fig. 4F; Supplementary File 2). RM.Gp1 preferentially expressed Fabp5, Ccl2, Ccl7 Ly6e, while RM.Gp2 preferentially expressed Saa3, Cd36, Spp1 & Timp1. RM.Gp2 were also identified as Cd11c positive unlike RM.Gp1 cells. A population of MoDM were also identified (Ccr2+, Apoe+) as H2-Ab1 positive, Itgax positive and SiglecF negative (RM.Ch-). This population had low expression of the AAM markers Chil3 and Mrc1 but had high levels of the AAM marker Retnla. A similar population of H2-Ab1 positive, Itgax positive and SiglecF negative recruited macrophages was also identified but had high expression of Chil3 (RM.Ch^+^). Two final populations of MNP cells were identified as alveolar macrophages based on positive expression of SiglecF and Cd11c, with one population displaying high expression of antigen presentation genes (AM.Apc) such as H2-Ab1 and the other not (AM) (Fig. 4D). As potential mediators of DEP enhanced type II inflammatory effects, mRNA levels for Ccl8 and Ccl24 were examined across these MNP subtypes (Fig. 4E, G). The expression of these genes did not overlap with the highest expression of Ccl8 found in RM.Mo, RM.Gp1 & RM.Gp2 cells, while the highest expression of Ccl24 was found in RM.Ch and AM.Apc cells. Interestingly, while expression of the AAM marker Arg1 was similar across RM, the AAM marker Retnla was preferentially expressed in a pattern across cell types that paralleled Ccl24 expression (Supplementary Figure S5B). The monocyte chemoattractant Ccl2 followed a pattern similar to Ccl8 (Fig. 4G), while the chemokine receptor Ccr2 had the highest expression in RM.Mo and RM.Ch cells.

### Trajectory analysis identifies monocyte derived recruited macrophages as a source of RM.Gp populations

We next performed trajectory analysis to identify pseudotime dependent differentiation relationships between the MNP populations using the RM.Mo population as a starting point (Fig. 4H). Two branches from the root population were identified as RM.Gp1 and RM.Gp2 indicating both populations develop separately along different trajectories from recruited MoDM. RM.Gp2 cells then followed a trajectory to RM.Ch + and AM.Apc through to AM cells indicating a differentiation towards resident alveolar macrophages over time.

### Alterations in MNP cell types and cell type specific expression of AAM / T2 markers

We next examined the numbers of cells within each MNP population. RM.Mo and RM.Gp2 populations, and to a lesser extent AM, demonstrated increases with D + H treatment when compared to HDM alone (Fig. 4I). Increases in total cells expressing Ccl8 and Ccl24 were also observed for D + H v HDM (Fig. 4J). Investigation of MNP subpopulation specific expression identified RM.Gp2 cells with significantly higher Ccl8 mRNA expression in D + H compared to HDM (Fig. 4K). While RM.Gp2 and AM populations demonstrated increases in Ccl24 mRNA, this was not found to be statistically significant (Fig. 4K). Total mRNA for Arg1 was higher in D + H compared to HDM in RM.Gp2 and RM.Ch- cells (Fig. 4K) a trend also observed for Retnla but not as pronounced (Fig. 4K).

### High content imaging characterisation of LAC MNP population dynamics and expression of T2 chemokines Ccl8 and Ccl24

**Fig. 5:**
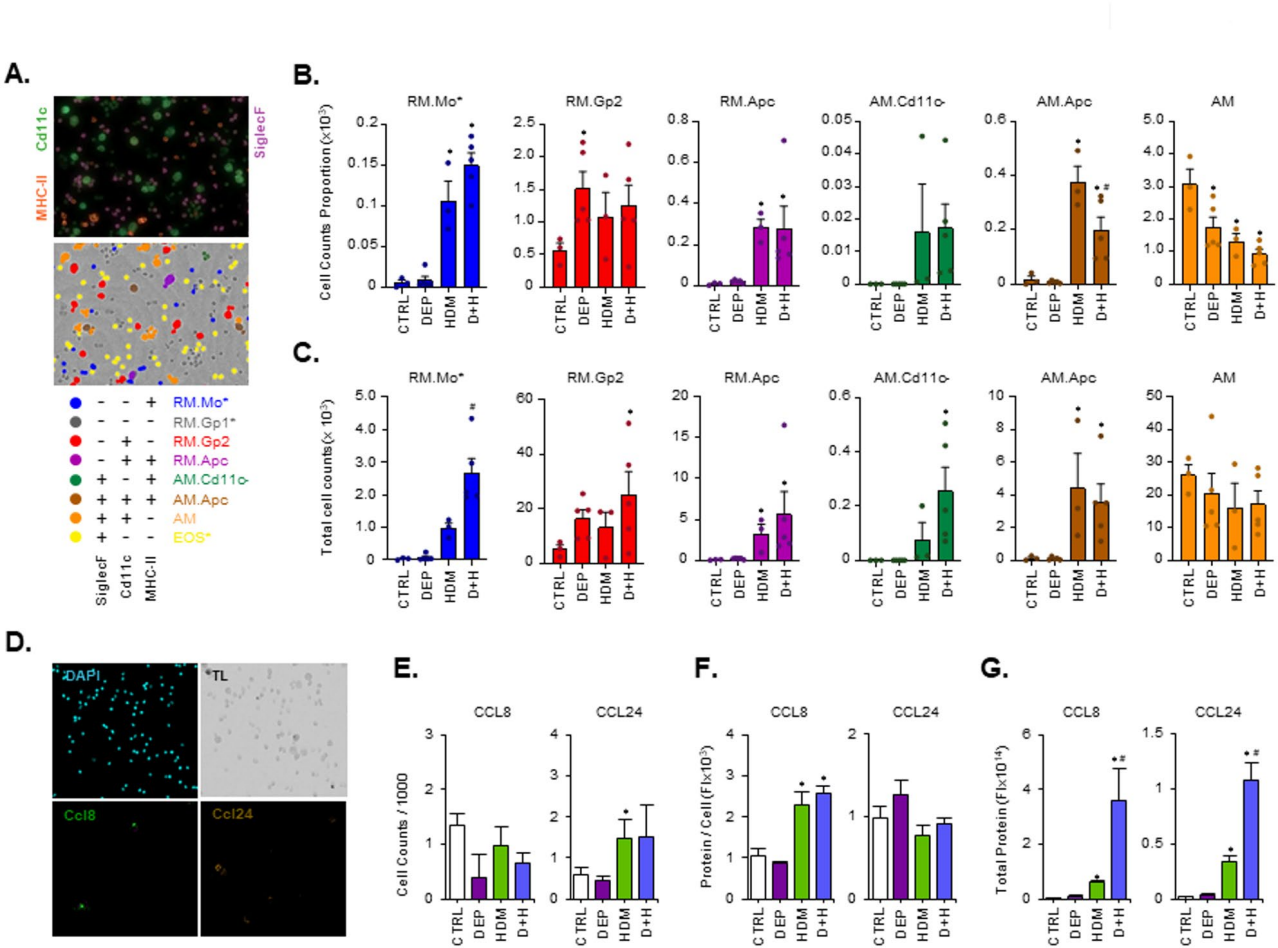
High content imaging analysis of LAC identifies DEP enhanced macrophage sub-population recruitment and increased chemokine expression. High content imaging was carried out on LAC isolated from CTRL (*n* = 3), DEP (*n* = 5), HDM (*n* = 3) and D + H (*n* = 5) repeat exposed mice (9 times over 3 weeks) immuno-fluorescently labelled for MNP markers (**A**-**C**) and chemokine expression (D-G). Cell types were identified with unique combinations of marker expression (A). Individual cell counts across CTRL, DEP, HDM and D + H treatments for each cell type is displayed as a proportion of 10,000 analysed cells per mouse (**B**) or normalised to total LAC MNP cells per lung (**C**). Imaging for Ccl8 and Ccl24 expressing cells is indicated (**D**), with DAPI nuclear stain and transmitted light (TL) reveal all cells in field of view (**D**). The numbers of chemokine positive cells per 1000 analysed is displayed (**E**). The fluorescent intensity (FI) was calculated as the sum of fluorescent intensity per pixel per positive cell and indicated as protein levels for chemokine expression per cell (**F**) and then adjusted for total LAC counts per lung (**G**). Results are expressed as Mean +/- SEM for each treatment group with individual data points indicated. Statistical comparisons of treatments are indicated as * (*p* < 0.05) when compared to CTRL levels and as # (*p* < 0.05), when compared to HDM

Using high content imaging detection of cell surface MNP protein markers MHC-II, Cd11c and SiglecF, we examined the number of LAC macrophage subtypes after DEP, HDM and D + H treatments (Fig. 5A-C). A population of cells containing RM.Mo displayed HDM dependent increases, which was further increased with DEP co-treatment (D + H) (Fig. 5B-C). As this population also likely contains B-cells, the specificity for RM.Mo responses from this observation cannot be confirmed. However, the markers used could specifically identify RM.Gp2 cells, which were proportionally increased within LAC when compared to control cells (Fig. 5B). While not statistically significant, both DEP and HDM alone showed increases in total RM.Gp2 cells, which was further increased to significant levels on combination (D + H) (Fig. 5C). HDM also increased RM.Apc, AM.Cd11c and AM.Apc total cells, which was further modified by DEP for AM.Cd11c^−^ only. There were no significant changes in total LAC AM (Fig. 5C). High content imaging was also used to examine LAC chemokine protein expression (Fig. 5D-G). As a proportion of examined cells, the number of Ccl24 expressing cells were significantly increased with HDM treatment but not by DEP, whereas Ccl8 expressing cells did not change (Fig. 5E). Examination of protein expression per cell demonstrated that HDM induced significant increases in Ccl8 levels, which was not altered by DEP alone or with co-treatment (Fig. 5F). No changes were observed for Ccl24 protein level per cell. Total protein content within the LAC compartment was also calculated and demonstrated significant increases in Ccl8 and Ccl24 with HDM treatment, which was further increased when combined with DEP (D + H) (Fig. 5G). DEP alone did not alter chemokine levels. To determine the location of Ccl8 and Ccl24 expressing cells within the lung we used RNAscope (Supplementary Figure S6C-D). Ccl8 and Ccl24 were both located in dense inflamed tissue surrounding blood vessels (Scgb1a1 negative), close to small airways. We used un-lavaged lungs to maximise the chances of observing LAC staining. While we did observe a small number of non-epithelial cells (negative for Scgb1a1) located in the small airway bronchiole lumen in D + H treatments but not in control treatments, we did not observe luminal airway cells with chemokine expression (Supplementary Figure S6C-D).

### Characterisation of luminal airway T-cell and NK-cell population changes on HDM and D + H treatment

**Fig. 6:**
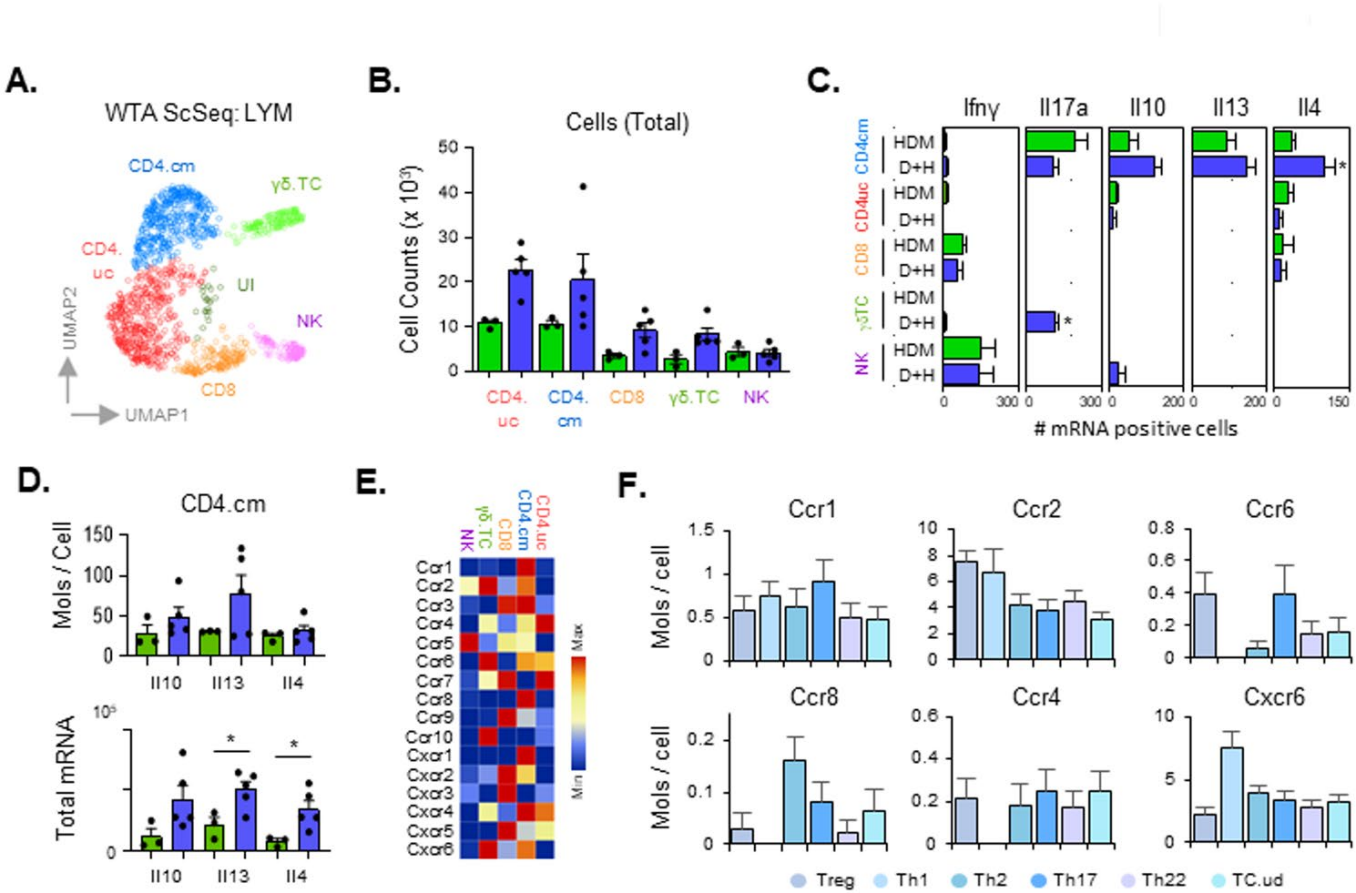
DEP enhances CD4 T-cell specific induction of type 2 cytokines Il4 and Il13. WTA Scseq analysis was carried out on LAC isolated from HDM (*n* = 3) and D + H (*n* = 5) repeat exposed mice (9 times over 3 weeks). T-cell and NK cell populations were identified (*CD4.cm* CD4 committed T-cell, *CD4.uc* CD4 Tcf7 positive uncommitted T-cell, *CD8* CD8 T-cell, *γδ.TC* γδ T-cell, *NK* Natural Killer cell, *UI* Unidentified) and visualised as a UMAP plot (**A**). Lung LAC total cell counts for these subpopulations are displayed (**B**). Those cells positive for helper T-cell (T_H_) cytokines for each cell type are displayed normalised to 1000 analysed (**C**). The number of mRNA molecules per cell for T_H_2 cytokines Il10, Il4 and Il13 (**C**; upper panel) and the total lung LAC mRNA levels (**D**; lower panel) within the CD4.cm population are shown. Graphic labels are green for HDM and blue for D + H exposures. Chemokine and Chemokine receptor levels across these cell types were also examined (**E–F**). The number of cells with positive expression for each gene was calculated for each cell type (per 1000 cells), normalised to the maximum counts for that gene across cell types and visualised as a heatmap (**E**). Select receptor expression was also examined across CD4.cm subtypes identified based on cytokine and transcription factor specific markers (**F**). Results are expressed as Mean +/- SEM for each treatment group. Statistical comparisons of D + H v HDM in WTA ScSeq data were carried out using unpaired student t-test with significance indicated as * (*p* < 0.05) or as specific p values

WTA single cell sequencing data was next examined for changes in T and NK cell populations which were positive for Cd3g/Thy1 and Nkg7 respectively (Supplementary Figure S4A), that may direct inflammatory responses in the LAC. In addition to NK cells, four main populations of T-cells could be identified (CD8, CD4.uc, CD4.cm and gδT cells) (Fig. 6A). Total cell numbers within the airway lumen for these cell types revealed non-significant increases in all T-cell sub-populations but not NK cells (Fig. 6B). Further examination of helper T-cell subtypes within the committed CD4 (CD4.cm) population identified Th1, Th2 and Th17 type cells. Ifng positive Th1 cells, were in low abundance and did not change between HDM and D + H treatments (Fig. 6C). Levels of Ifng in CD8 and NK cells were more abundant but did not show any change between treatments. T_H_17 cells (Il17a+) were present with HDM treatment but were reduced upon co-treatment with DEP (D + H). Interestingly Il17a positive gδT cells were absent with HDM alone but had significantly higher levels in D + H (Fig. 6C). Increases in Th2 cells were observed for Il10, Il13 and Il4 positive CD4 cells in D + H compared to HDM alone. Total mRNA levels within the LAC compartment demonstrated increased levels for these T_H_2 cytokines, reaching statistical significance for Il13 and Il4 (Fig. 6D), effects also observed using bulk-seq analysis (Supplementary Figure S7D). Examination of cell specific chemokine and chemokine receptor expression was carried out to provide insight into mechanisms of T-cell recruitment (Fig. 6E–F). CD4.cm cells containing memory T_H_ cell populations displayed higher expression of Ccr1, Ccr3, Ccr8, Cxcr1 and Cxcr4 than other types of T and NK cells (Fig. 6E). Subpopulations of CD4.cm cells were also examined (Fig. 6F) and demonstrated higher expression for Ccr2 in Treg and T_H_1 cells, higher expression of Ccr8 in T_H_2 cells and higher expression of Cxcr6 in T_H_1 cells.

### DEP priming facilitates increased F4/80 macrophage responses to T2 cytokine exposure

**Fig. 7:**
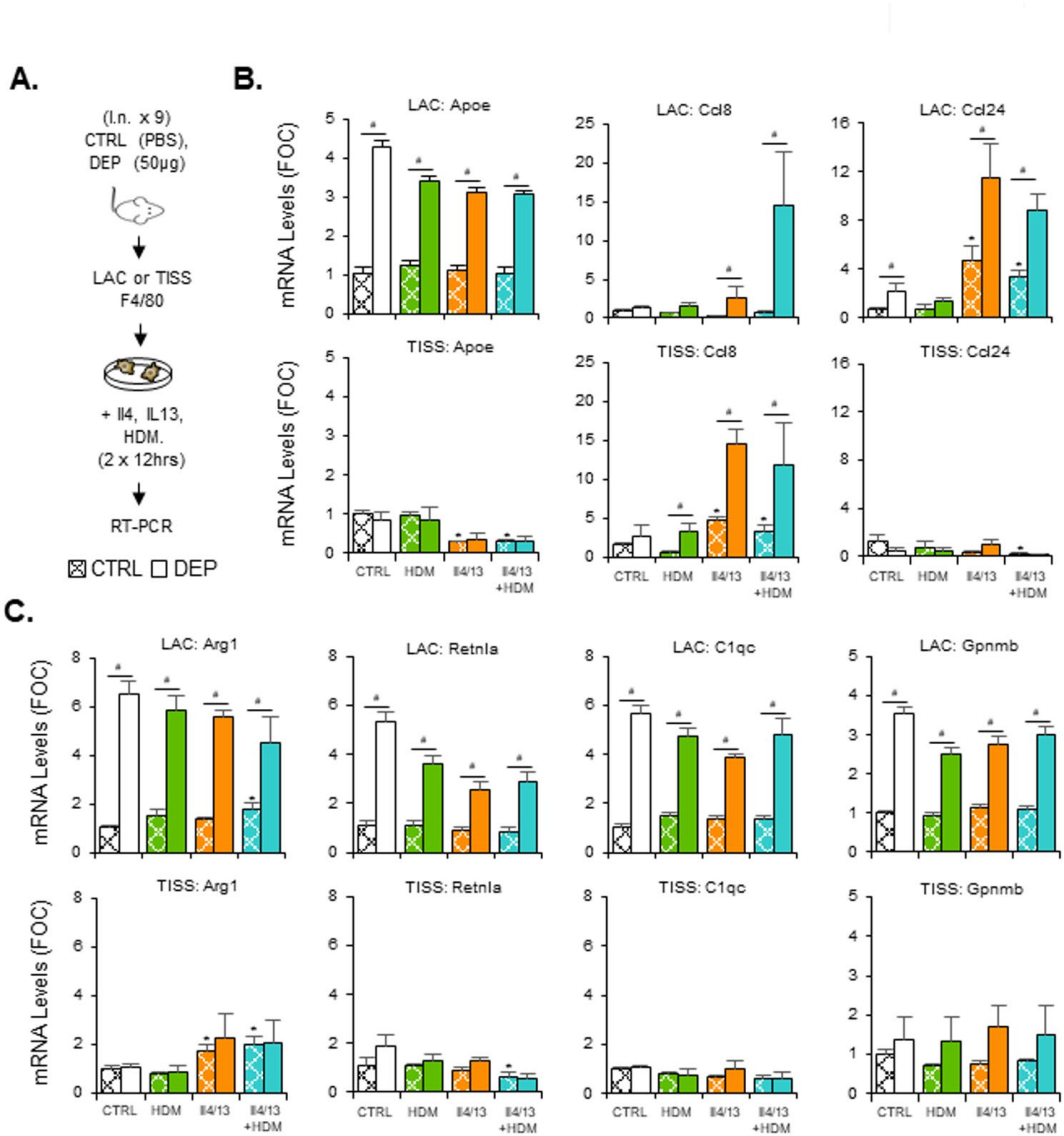
DEP pre-treatment enhances LAC F4/80 macrophage production of Ccl8 and Ccl24 in response to Il4 + Il13. Mice were intranasally exposed to either CTRL (PBS) or DEP (x9 repeats over 3 weeks, n = 4 per group). F4/80 positive macrophages were then isolated from either the LAC or TISS compartment using antibody based immunomagnetic separation. Cells were then cultured ex vivo on low attach plates and exposed to HDM (25 ug/ml) and Il4 + Il13 (10 ug/ml) either alone or in combination (**A**). mRNA was isolated after two sequential exposures (12 h each) and analysed for chemokine (Ccl8, Ccl24) and macrophage (Apoe, Arg1, Retnla, C1qc, Gpnmb) marker gene expression by RT-PCR (**B**, **C**). Fold over control (non-DEP pre-treated cells) levels (FOC) were calculated and results expressed as Mean +/- SEM for each treatment group. FOC levels were calculated within LAC and TISS compartments separately. Results are expressed as Mean +/- SEM for each treatment group. Statistically significant comparisons are indicated as * (p < 0.05) for ex vivo treatments within the non-DEP in vivo pretreated group, while # (p < 0.05) indicates significance between DEP pretreated versus non-DEP for each ex vivo treatment

With observations that DEP enhanced HDM induced Il4 and Il13 expression in CD4 T-cells (Fig. 6) and that their receptors are highly expressed on LAC MNP cells (Supplementary Figure S7A) we next investigated whether these cytokines could modify macrophage production of Ccl8 and Ccl24 in DEP pre-treated mice in both LAC and TISS compartments (Fig. 7). F4/80 (Adgre1) expressing cells (Supplementary Figure S7B) were isolated from mice after CTRL or DEP instillation and incubated in vitro with combinations of Il4, Il13 and HDM, prior to gene expression assessment after 24 h (Fig. 7A). The RM marker Apoe was increased with DEP in the LAC but not the TISS compartment with a modest reduction in LAC mRNA levels with in vitro exposures (Fig. 7B). This pattern was repeated for the AAM markers Arg1, Retnla, the IM marker C1qc and the RM.Gp cell marker Gpnmb (Fig. 7C). Ccl8 expression in the LAC compartment was increased with Il4 + Il13 treatment in the DEP pretreated samples only, an effect not observed with HDM alone (Fig. 7B). LAC from mice that did not receive DEP pre-treatment did not show any changes in Ccl8 with in vitro exposures. On combination (Il4 + Il13+HDM) there was enhanced expression of Ccl8 compared to Il4 + Il13 in LAC. Within the TISS compartment of CTRL pretreated mice, Ccl8 levels were increased with IL4 + Il13 but not HDM. DEP pretreatment significantly enhanced Ccl8 levels of all in vitro treatments but there was no difference between Il4 + Il13 versus Il4 + Il13+HDM (Fig. 7B). For Ccl24 in the LAC compartment, DEP pretreatment enhanced Il4 + Il13 induced levels but again there was no difference between Il4 + Il13 versus Il4 + Il13+HDM. Within the TISS compartment for Ccl24, there was a significant decrease in expression with Il4 + Il13+HDM, which was not modified by DEP pretreatment (Fig. 7B). We attempted to validate these gene expression changes at the protein level, but Ccl8 and Ccl24 concentrations in the macrophage culture supernatants were below assay detection limits (likely due to the low cell numbers per well in a relatively large volume of cell culture media, coupled with a lack of sensitivity of the ELISA assay we used). Notably, in vivo BAL fluid protein measurements in our study (Fig. 3H) showed that changes in Ccl8/Ccl24 mRNA correspond to increased protein levels, supporting that the observed mRNA upregulation in F4/80^+^ macrophages translating to functional protein production.

### Endotoxin levels across treatment preparations

Endotoxin activity in house dust mite allergen preparations is an important component responsible for mediating inflammatory responses in the lung, including allergic sensitisation and local airway type 2 inflammation [47, 48]. As particulate preparations of DEP and CEP may contain levels of LPS (endotoxin) responsible for enhanced effects of particles on HDM induced airway inflammation, we measured levels of endotoxin using a chromogenic Limulus amoebocyte lysate (LAL) assay. Levels of endotoxin in DEP and CEP particles were not significantly different from control PBS levels, while HDM preparations displayed significantly higher levels (Supplementary Figure S7C).

## Discussion

The main aim of this study was to characterise and identify cellular and molecular drivers underpinning the enhancement effects of DEP on HDM induced allergen induced inflammation including type 2 responses associated with allergic airway disease. Initially we used TGE ScSeq analysis, which provided a high degree of granularity in identifying many different cell types within the luminal airway compartment with D + H exposure, compared to mainly macrophage and monocyte populations present with CTRL treatments only. Cell type specific markers were identified from TGE and the wider literature and used for cell interpolation of bulk-seq datasets across LAC and TISS compartments. We observed a much larger transcriptomic response to treatments within the LAC compared to TISS compartments, which we suggest is due to a greater change in the cellular composition of the LAC compartment than TISS and not due to differences in sensitivity or variance between sample types. This predominant luminal response to respiratory challenge has been observed in other studies. Segmental instillation of LPS into human airways induced thousands of transcriptomic changes in gene expression in LAC, while only 1 gene was differentially expressed in corresponding paired mucosal biopsies [49]. Similarly, HDM pulmonary exposure in mice was found to have greater proteomic changes in the BAL fluid when compared to corresponding lung tissue [50]. These observations taken together with our study findings, including our RNAscope data identifying non-epithelial cells within the conducting airway lumen in D + H treatments, highlights how the luminal airway compartment may be an under-appreciated immunologically active site, responsible for co-ordinating DEP’s ability to enhance HDM induced allergic airway inflammation.

Interrogation of bulk-seq and cell interpolation data revealed a larger response to HDM than DEP in LAC, an effect also observed in TISS but not to as great an extent. The administered mass dose of DEP is twice that of HDM indicating HDM a more potent exposure material than DEP. In the TISS compartment, the greatest enhanced response (D + H over either DEP or HDM alone) was observed for mast cells and secretory epithelial cells, consistent with their established location in mucosa. The effects in LAC were more diverse with enhanced D + H effects most prominent for AAM, B cells and eosinophils. The extent to which a particular cell type was the causative mechanism for enhanced effects or as an effector cell of the resulting immune response, or both, was initially unclear. To investigate this further we looked to compare to another particle type with the ability to enhance allergen induced type 2 inflammation. We have previously demonstrated that another pollutant nanomaterial CEP, with similar size distribution, had near identical T2 enhanced activity for HDM in this same 3 week exposure protocol [41]. A comparison of LAC bulk-seq data from both material exposures (D + H v C + H) revealed the most common cell type associated with particle enhancement effects as AAM (Arg1, Timp1, Chil3, Ccl24). These cells are key coordinators of tissue repair after injury, and type 2 inflammation in allergic airway disease, with their differentiation primarily driven by the T2 cytokines IL-4 and IL-13 [51, 52]. The alarmin IL-33 has also been observed to drive AAM differentiation [53] but is unlikely to play a role in LAC AAM differentiation in our study as no alterations in this cytokine were found in the luminal compartment. In addition to AAM marker expression enhanced in D + H and C + H exposures, the chemokine Ccl8 was also highly enhanced compared to particle or HDM alone treatments. While not typically identified as an AAM marker, Ccl8 was observed as a T2 cytokine dependent produced from Cd11c^+^ inflammatory macrophages in a mouse model of early life HDM induced allergic airway disease [15]. These Cd11c^+^ cells originate from recruited Ccr2^+^ monocytes and displayed mixed population characteristics of both classical (Nos2) and alternatively activated (Chil3) macrophages. Blockade of the Ccl8 receptor Ccr8 prevented T_H_2 and ILC2 cell recruitment in this model [15]. In our own study we also observed selective expression of Ccr8 on T_H_2 cells and with our observations that combined exposure (D + H) enhanced Ccl8 levels and T_H_2 cells, we suggest this chemokine as an important regulator of the enhancement effects of DEP on HDM induced T2 inflammation. Furthermore, enhanced expression of Ccl24 and Ccl2 with combined exposure (D + H) over HDM levels, we suggest also contributes to DEP enhanced T2 inflammation in the LAC compartment, through the recruitment of Ccr3^+^ eosinophils and Ccr2^+^ monocytes respectively.

Within the luminal airway compartment these D + H enhanced chemokines are suggested to originate from MNPs [15, 54, 55]. We used WTA ScSeq to examine the profile of MNPs altered by HDM and to investigate which population of cells are responsible for DEP enhanced effects. We identified Cd11c^+^ (Itgax), SiglecF^−^, H2-Ab1^−^ recruited macrophages increased in D + H versus HDM in the LAC. This cell type termed RM.Gp2 matches Cd11c^+^ inflammatory macrophages identified previously as the source of Ccl8 in a model of AAD [15]. Importantly, we identified these cells as the only population with increased Ccl8 induced by DEP co-exposure (D + H) when compared to HDM alone. While RM.Gp1 cells also had high levels of Ccl8 in HDM, there was no increase in Ccl8 with D + H treatment in this population. D + H also increased RM.Mo cells, a population of cells with properties of newly recruited Ccr2^+^ monocytes but modified by the T2 environment indicated by the presence of AAM marker Arg1 and APC markers, H2-Aa and Cd74. Examination of cell profiles at earlier time points after exposure, as opposed to 24 h after the last pulmonary exposure used in this study, would likely reveal these newly recruited Ccr2^+^ monocytes without further macrophage differentiation markers. Nevertheless, as trajectory analysis of Scseq data revealed differentiation of RM.Gp2 cells directly from RM.Mo, we suggest that an increase in RM.Gp2 cell numbers in D + H is due at least in part to increased recruitment of Ccr2^+^ monocytes. It follows that this increased pool of RM.Gp2 cells likely also contributes to the increased Ccl8 expression in D + H versus HDM. The transient nature of monocytes when recruited to active inflammatory sites is well established [56, 57], where they rapidly differentiate into macrophage subtypes dependent on local environmental cues. This explains the relatively low numbers of RM.Mo cells identified in our single cell sequencing and high content image analysis in the LAC compartment compared to other myeloid cell types. Examination of cell recruitment to the BAL earlier than 24 h used in our study would likely reveal a recognisable pool of undifferentiated monocytes before differentiation to RM subtypes. The recruitment of Ccr2^+^ classical monocytes is driven primarily by its ligand Ccl2 [58], which we identified as selectively expressed in RM.Mo and RM.Gp1 cells. While we did not observe enhanced Ccl2 in Scseq data, which may be a consequence of differences in sensitivity or method specificity, we did observe its increase in the LAC at protein and bulk-seq mRNA level and suggest that Ccl2 mediated Ccr2^+^ monocyte recruitment is critical for DEP enhanced effects. Indeed, it has been demonstrated that in Ccr2 KO mice, DEP was unable to enhance allergen induced allergic airway disease when compared to WT controls [20]. Our WTA Scseq results indicate enhanced Ccl24 in the LAC results from a culmination of expression across different RM (including RM.Gp2) and AM cell types.

Confirmation of DEP enhanced HDM induced increases in LAC RM.Mo and RM.Gp2 cell population numbers was also observed using high content imaging. This analysis indicates that while proportionally HDM induced changes are minimally increased by DEP co-exposure, when adjusted for macrophage counts, total cell numbers for these RM populations within the LAC compartment are significantly increased. Similarly, Ccl8 and Ccl24 positive cells and protein levels in each cell were proportionally unaltered between HDM and D + H, but when adjusted for total LAC macrophage content per lung, the increases in protein were significantly altered. These data support the hypothesis that increased total monocyte and derived macrophages in the luminal airway compartment contribute to the increases in Ccl8 and Ccl24 chemokine expression.

Of further interest was the observation that Ccl8 and Ccl24 displayed near exclusive non-overlapping cell type expression, which may reflect inherent differences in cell responsiveness, or may be a consequence of cells experiencing different spatial-temporal influence as they are differentially located across the luminal airway compartment. Parallel to these findings was the observation that the AAM markers Arg1 and Retnla did not have the same cell type dependent expression and that cells expressing Ccl8 were more likely associated with Arg1 (RM.Gp1, RM.Gp2) and cells expressing Ccl24 were more likely associated with cell populations expressing Retnla (RM.Gp2, RM.Ch^−/+^, AM.Apc). These data suggest that the previously held definition of AAM as a single cell type or cell state may be better described as a collection of different sub-populations with different origins that may explain the cell diversity in marker expression. Indeed, while T2 cytokines are considered the main drivers of the AAM phenotype, within the lung it is unclear how resident and recruited MNPs contribute to the AAM pool [59]. A recent study of allergen induced airway disease demonstrated that T_H_2 cell derived IL-4 & IL-13 was responsible for luminal airway AAM development primarily from AM, but that a smaller population also from newly recruited Ccr2^+^ monocytes [60]. Indeed, monocytes are considered more highly responsive for differentiation to AAM compared to AM [61], again supporting the premise that different AAM populations may develop during injury and disease. Another recent study has also identified different AAM populations in a model of muscle injury resolution in mice. Retnla^+^ AAM derived from resident macrophage populations, expressed Ccl24 which was responsible for recruitment of eosinophils into injured tissue. Arg1^+^ AAM, which also expressed Spp1, derived from newly recruited Ccr2^+^ monocytes and were responsible for clearance of debris material and the regeneration of injured tissue [62]. These same Arg1^+^ and Retnla^+^ AAM cell types were identified in our study, which we suggest also originate from Ccr2^+^ monocytes and AM respectively. Despite Ccl24 expression most highly expressed on a per cell basis in RM.Ch and AM.Apc, DEP enhanced total Ccl24 derives from not only the RM.Gp2 population but also the AM.Apc and AM populations. With DEP enhanced Ccl8 only arising from the Arg1^+^ RM.Gp2 population, these observations indicate separate but parallel chemokine programs in different luminal airway AAM macrophages that are responsible for DEP enhanced HDM allergic inflammation.

The gene expression profile of Gpnmb^+^ recruited macrophage populations (RM.Gp1 and RM.Gp2) in our study (Supplementary Figure S5G) demonstrated striking similarity to newly described monocyte derived Spp1+/Gpnmb^+^ macrophages, which also express Trem2, Fabp5, Cd63 and Apoe, and are found increased in different disease states and injury models [46, 63–65]. It was initially suggested that these cells played a role in driving fibrosis in humans and murine models of disease [46, 65]. However, further study revealed that while these cells were associated with fibrotic injury, they acted in a pro-resolution capacity [45]. Another study also demonstrated their presence in a bleomycin induced model of pulmonary fibrosis but through comparison with LPS induced pulmonary inflammation, where these cells were also recruited, suggested that while these cells are necessary for fibrotic mechanisms, they are not sufficient on their own [43]. These cells have interestingly been identified recently in a model of HDM induced AAD in mice, where they are suggested to function as pro-resolving M2 like cells and also expressed the AAM markers Arg1 and Chil3 [66]. These AAM signatures were not observed on Spp1/Gpnmb cells in non-allergen disease or injury states. These observations therefore suggest that RM.Gp cells identified in our study are the same Spp1^+^/Gpnmb^+^ macrophages identified in these other studies. We further suggest that these cells display context specific additional functionality in response to local stimuli, in our case, adopting an additional profile of AAM gene expression and functionality as part of the development of allergic airway diseases (AAD) and T2 inflammatory processes. Furthermore, we suggest that in addition to HDM recruitment, DEP exposure increases the pool of these macrophages in the airway lumen as a mechanism of its enhanced effects on HDM induced allergic type 2 inflammation.

The increase in AAM markers and T2 associated inflammatory endpoints with D + H treatment over HDM suggest the presence of enhanced T2 driver cytokines IL-4 and / IL-13 and the cells that produce them. Indeed, we demonstrate increased protein levels in the luminal airway compartment. Typically, these cytokines arise from T_H_2 or ILC2 cells. We observed very few (TGE ScSeq) or no (WTA ScSeq) ILC2 cells within the LAC but did find a large number of T_H_2 cells within the committed CD4 T-cell population (CD4.cm), expressing IL-4, IL-10 or IL-13. DEP enhanced levels of these cells and T_H_2 cell specific cytokines in our study are consistent with previous work demonstrating that T_H_2 cells and not ILC2 cells contribute to DEP enhanced HDM AAD [67]. The recruitment of T-cells to the luminal airway (LA) compartment is an important step in the initiation and maintenance of T2 inflammation. We observe a complex pattern of chemokine receptor expression across different populations of T-cells. Of note, was the expression of Ccr2 on CD4.cm and gδT cells, but not other T-cell types, indicating an active role in effector T-cell recruitment. Ccr2 levels were also comparable to levels found in Ccr2^+^ recruited macrophages. It may be suggested that enhanced Ccl2 with D + H over HDM may contribute to the initial recruitment of effector Ccr2^+^ T-cells and play a functional role in DEP adjuvancy. Ccr2 has been previously observed as expressed on effector T-cell populations [68] and while it remains to be proven, Ccr2 mediated T-cell recruitment may be considered as a contributing mechanism of DEP adjuvancy in KO studies, where Ccr2 is universally eliminated from all cell lineages, not just monocytes where most of the attribution of function is given in these studies [20]. We also observe the Ccl8 receptor Ccr8 was more highly expressed on T_H_2 than any other effector T-cell, consistent with this pathway playing a role in T_H_2 cell recruitment.

Ex vivo exposure of DEP primed LAC F4/80 macrophages to IL-4 + IL-13 increased the expression of Ccl8, an effect not observed in CTRL LAC cells. HDM co-exposure increased levels further but alone did not have any effect. Increased Ccl24 followed a similar pattern but was not enhanced by HDM exposure. DEP populations displayed increased RM and AAM markers (Apoe, Arg1, Retnla, Gpnmb) compared to CTRL. These observations are consistent with T2 cytokines as the main driver of Ccl8 and Ccl24 expression from DEP newly recruited macrophages. Ccl24 increases by IL-4/13 were observed in CTRL cells, unlike Ccl8, and are consistent with our previous observations and suggestion that Ccl24 responsiveness is not restricted to RM but also involves resident AM populations. Corresponding mucosal tissue responses to IL-4/13 demonstrated DEP primed increases in Ccl8, with no impact on Ccl24 and indicate further complexity of macrophage response to T2 and allergen exposures in this compartment.

We have demonstrated that DEP enhances the number of HDM induced monocyte derived recruited macrophages, thereby increasing the AAM pool of responsive cells to T2 cytokine stimulation, which we suggest then co-ordinates the recruitment of type 2 inflammatory cells, through enhanced chemokine expression. In addition, we observe enhanced numbers of effector T cells and derived T2 cytokines with DEP, attributable to RM derived Ccl8. Other chemokine recruitment mechanisms such as Ccl2-Ccr2 and Ccl17-Ccr4 may also be involved as each were found enhanced by D + H over HDM in our datasets. However, unlike Ccr2, which was expressed on effector T-cells, Ccr4 was mainly expressed on uncommitted T-cells and did not display any selective expression on T_H_2 cells. Interestingly, a population of GATA3^+^ TCF7^+^ multipotent progenitor T_H_2 cell (T_H_2-MPP) type has recently been identified in human allergic airway tissue, selectively expressing CCR4, compared to effector T_H_2 cells. These cells act as a pool of TSLP responsive stem cells capable of differentiation into cytokine producing T_H_2 effector or other types of T-cells to propagate allergic inflammation [69]. It is plausible that the CD4.uc population in our study contains these TH2-MPP cells and that Ccl17-Ccr4 pathway acts to recruit these cells which then locally expand in response to TSLP to increase effector TH2 cells. Indeed, we observe increases in TSLP on combined D + H exposure in the LAC, not observed with either treatment alone.

Evidence has accumulated demonstrating a role for many different cell types, including DCs, epithelial cells, T-cells and neutrophils [67, 70–75] in the enhanced effects of pollutant particles in allergic airway disease. Furthermore, it is clear that as well as intrinsic biological activities of allergen proteins including protease activity, pattern recognition receptor (PRR) ligands such as LPS, chitins, and β-glucans also contribute to allergen induced allergic airway disease and inflammatory responses [76]. We tested whether LPS (endotoxin) contamination of DEP or CEP particles could be responsible for particle enhanced HDM effects but found no increased levels when compared to control. We cannot however rule out other PRR ligands as contributing factors to enhanced inflammatory effects. While the LAL assay used in this assay may also detect β-glucans, the likelihood protease or chitin contamination of particles used in this study is low as they are more typically found in environmental samples, as opposed to LPS which is more commonly found in combustion or purified nanomaterial preparations. It has also been suggested that rather than having a direct impact on antigenicity or antigen presentation, pollutant particulates increased allergen immunogenicity due to slower degradation and longer persistence of altered antigens thereby increasing DC activation [77]. This is postulated to occur as part of the formation of a “corona” of allergens on particle surfaces. For example, carbon nanotube enhancement of HDM induced eosinophilia and T2 airway inflammation was attributed to accumulation of allergens on the surface of the particle, allowing more potent activation of dendritic cells [78]. While testing this possible mechanism was beyond the scope of this current study, we recognise it as a possible contributor to particle enhancement of HDM induced type 2 inflammation. These studies attest to the presence and complexity of multiple mechanisms and multicellular processes, that may all contribute sequentially and / or in parallel across different locations within the airways and at different time scales, to progress the development AAD. Our work adds to this body of knowledge and highlights DEP enhanced monocyte derived macrophages within the airway space as potential central players in AAM development, chemokine enhancement and recruitment of T2 inflammatory cells. Additionally, we suggest that RM.Gp cells identified in our study, also identified as Spp1/Gpnmb cells in other studies of injury and disease, may exist as a primed checkpoint or facilitator population recruited in many adverse tissue conditions, with the plasticity to respond to different environmental cues and guide appropriate inflammatory responses.

It is important to note the limitations of our study. Our observations indicate potential mechanisms of enhanced allergen induced type 2 inflammation by pollutant particle co-exposure. However, any causative roles for cells, cellular differentiation trajectory or mediators in overall disease outcome cannot by asserted at this point, as we did not carry out any lineage assessment or molecular interventional studies. Our study used female balb/c mice as a well-established model of HDM induced type 2 inflammation. While female mice are more susceptible to developing allergic airway inflammation [79] and therefore a useful sensitive model to carry out this work, we acknowledge that effects may not be the same in male mice, and therefore for a more complete assessment of disease effect, future exploration of these observations should incorporate both sexes into the study design. The doses used in our study are considered high when compared to typical human exposure scenarios. They were chosen based on established work in the field and to maximise the capability to detect important cellular and molecular alterations underpinning inflammatory events. While these levels may be reached at high deposition sites such as carina airway bifurcation points on particle inhalation [80, 81], it is uncertain whether the same effects observed in our study, would be seen at lower, more typical human inhalation exposure levels. Future work should explore these uncertainties.

## Conclusions

**Fig. 8:**
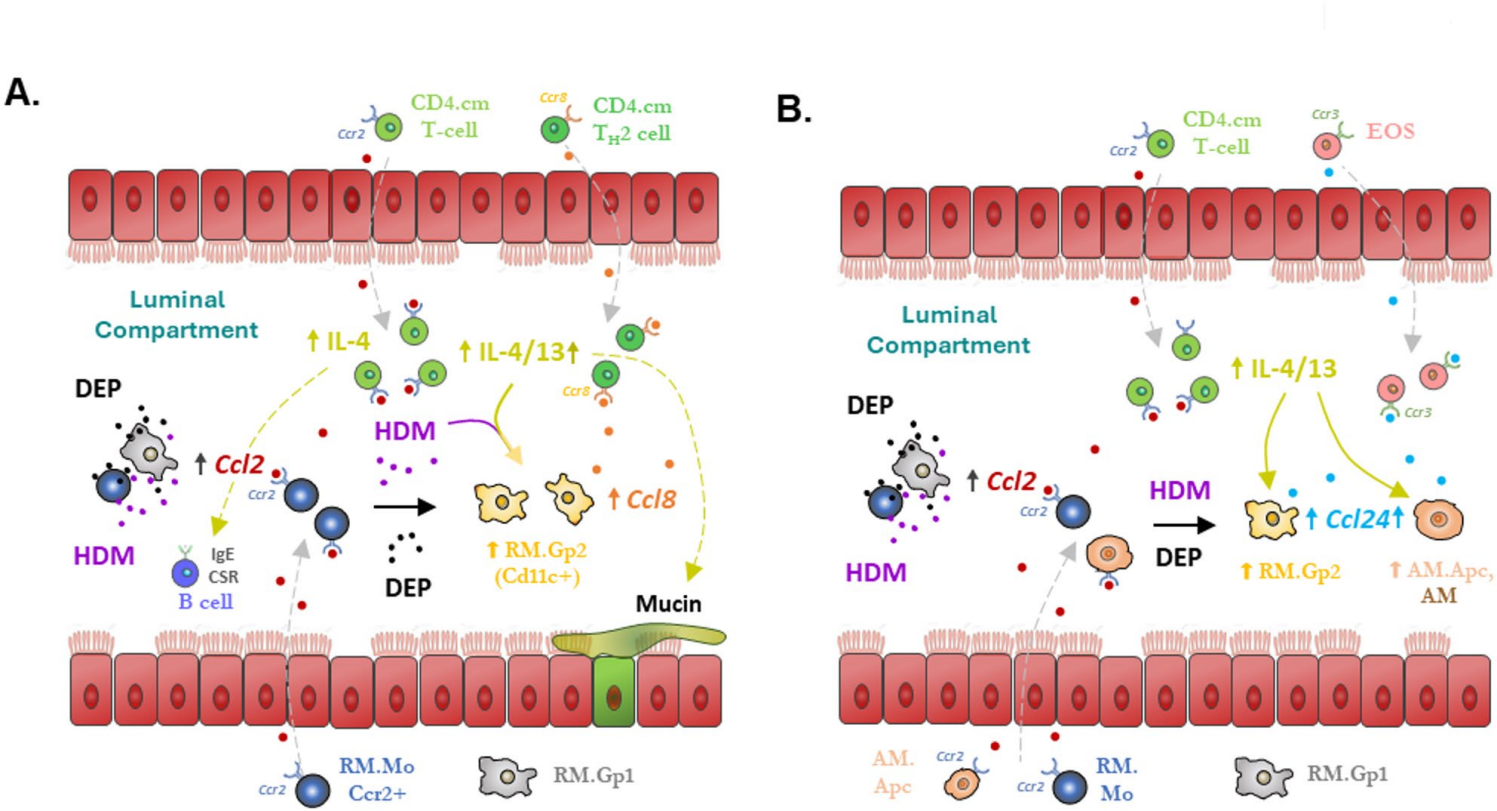
Suggested mechanisms underpinning DEP enhanced HDM induced type 2 inflammation. **A**. DEP and HDM co-exposure increase Ccl2 expression from luminal airway myeloid cells including RM.Mo and RM.Gp1 cells. Ccl2 attracts Ccr2+ RM.Mo cells to the airway lumen where they differentiate to RM.Gp2 cells. DEP and HDM independently increase cell numbers and or differentiation to RM.Gp2 cells. Ccr2+ CD.cm T cells also accumulate in the lumen due to DEP enhanced HDM induced chemokine (e.g. Ccl2) expression, resulting in increased release of IL-4 and Il-13 from TH2 cell subtypes. These type 2 cytokines act specifically on RM.Gp2 cells in conjunction with HDM to increase the Ccr8 ligand and chemokine Ccl8, which further attracts TH2 CD.cm T-cells to the airway lumen. Enhanced IL-13 and / or IL-4, may then increase type 2 inflammatory responses such as mucin production and B cell class switching recombination (CSR) to IgE. **B** Similarly, DEP enhanced HDM induced Ccl2 expression from myeloid cells increase AM.Apc cells alongside RM.Mo and RM.Gp2 cells, which together with a proportion of AM cells respond to enhanced luminal type 2 cytokines IL-4 and IL-13 to increase expression of the Ccr3 ligand and chemokine Ccl24. Increased levels of Ccl24 attract Ccr3 expressing eosinophils as a further mechanism of DEP enhanced type 2 inflammatory effect

This study examines how diesel exhaust particles interact with house dust mite allergen to amplify allergic airway inflammation at the exposure mucosal interface. Our data suggest the airway lumen as an important setting in which particles intensify allergen responses. We find that DE particles alone have modest effects, but in combination with HDM allergen they reshape the luminal compartment, enlarging a monocyte-derived macrophage pool (RM.Gp cells) likely recruited through the Ccl2-Ccr2 axis and sensitising it to type-2 cytokines, which are also increased in parallel. Subsequent production of the chemokines Ccl8 and Ccl24 from RM and AM populations, we suggest act to recruit T_H_2 cells and eosinophils, providing a coherent mechanism for pollutant enhancement of T2 inflammation. A graphical summary of these findings is provided (Fig. 8). The same organisational logic appears with a compositionally distinct material of the same size (CEP), indicating a shared mechanism may exist across different particle types. It follows that a shared property or properties of these particles is responsible. CEP lack the combustion associated metallic and organic components present on DEP yet still augment HDM responses, suggesting that these DEP properties are not the dominant drivers in this context. Rather, a common physicochemical attribute of ultrafine particles such as size, surface reactivity, surface area or coronal antigen binding is likely key.

The wider implications and impact of these findings include a better understanding of exposure hazard and environmental inhalation interactions, and the ongoing hazard air pollution poses to susceptible individuals. This work also supports the generation of foundational data to improve mechanistic frameworks of adverse effect understanding (e.g. adverse outcome pathways) and allow for appropriate testing of particle types and properties in appropriate and higher throughput methods including in vitro models, in ways not practically achievable solely using in vivo approaches. Identification of pollutant properties of highest hazard potential is an essential step towards the most effective mitigation strategies.

Taken together, these findings provide a compartment-resolved map of how pollutant particles modify allergen driven inflammation in the lung. While causality cannot be inferred from the present data, the convergence of bulk transcriptomics, single-cell profiling and protein-level validation prioritises monocyte derived recruited macrophages and their chemokine programmes as plausible amplifiers of type 2 inflammation. Importantly, this suggested mechanistic pathway does not exclude parallel contributions from epithelial responses (e.g. Alarmins), neutrophil heterogeneity, or dendritic cell/T cell crosstalk, which remain to be mechanistically tested in future interventional studies.

## Supplementary Information

Below is the link to the electronic supplementary material.

Supplementary Material 1.

Supplementary Material 2.

Supplementary Material 3.

Supplementary Material 4.

## Data Availability

The datasets used and/or analysed during the current study are available from the corresponding author on reasonable request. Transcriptomic datasets will be deposited with the ENA and ArrayExpress repositories.

## Abbrevations

+: Positive for expression
+ve: Positive
AAD: Allergic airway disease
AAM: Alternatively activated macrophages
Abseq: Antibody-oligonucleotide conjugate single cell sequencing
AM.Apc: Antigen presenting alveolar macrophages
AM.Cyc: Cycling alveolar macrophages
AM.Mt2: Mt2 expressing alveolar macrophages
AM: Alveolar macrophages
BAL: Bronchoalveolar lavage
BAS: Basophils
C+H: CEP + HDM
CB: Carbon black
CD4.cm: Committed CD4 T cells
CD4.uc: Uncommitted CD4 T cells
cDC2: Conventional dendritic cells type 2
CEP: Cerium dioxide nanoparticles
CTRL: Control
D+H: DEP + HDM
DCs: Dendritic cells
DEGs: Differentially expressed genes
DEP: Diesel exhaust particles
DES: Differential expression score
EOS: Eosinophils
gdT cells: Gamma delta T cells
HDM: House dust mite
HPMC: Human primary myeloid cells
IM: Interstitial macrophages
LA: Luminal airway
LAC: Luminal airway cells
LPS: Lipopolysaccharide
LYM: Lymphocytes
MAC: Macrophages
MC: Mast cells
MNP: Mononuclear phagocytes
MoDM: Monocyte derived macrophages
Mols: Molecules
NEU: Neutrophils
NHBAL: Normal human bronchoalveolar lavage
OVA: Ovalbumin
PBS: Phosphate buffered saline
pDC: Plasmacytoid dendritic cells
PM: Particulate matter
PMNs: Polymorphonuclear leukocytes
RM: Recruited macrophages
RM.Ch: Chil3 expressing recruited macrophages
RM.Gp: Gpnmb expressing recruited macrophages
RM.Mo: Recruited macrophages, monocyte like
RPKM: Reads per kilobase per million
RT-PCR: Reverse transcriptase – polymerase chain reaction
ScSeq: Single cell sequencing
SEM: Standard error of the mean
T2: Type 2
TGE: Targeted gene expression
TH1: Type 1 helper T cells
TH17: Type 17 helper T cells
TH2: Type 2 helper T cells
TISS: Tissue
UMI: Unique molecular identifier
WTA: Whole transcriptome analysis
