# Supplementary material for: Pollutant particles enhance house dust mite induced type 2 inflammation and the recruitment of monocyte derived Cd11c+ Gpnmb+ macrophages to the airway lumen
Source: Part Fibre Toxicol. 2026 Apr 11;23:23. doi: 10.1186/s12989-026-00675-8 (PMC13126997; doi:10.1186/s12989-026-00675-8)
Supplement: Supplementary file 2 — Supplementary Material 2. [file 12989_2026_675_MOESM2_ESM.docx]

**Supplementary Methods**

***RNAscope for mRNA levels in Lung tissue***

RNAscope was performed using Advanced Cell Diagnostics Hiplex12 Reagent Kit v2 (480, 550, 650) assay reagents and kit (ACD, CA, USA) (Cat # 324443 & Cat # 322380). All procedures were carried out according to the manufacturer’s instructions with the following modifications. For the fresh-frozen tissue sample preparation, slides were prepared in Optimal Cutting temperature (OCT) media (Tissue_Tek, Sakura Finetek, USA) as 15 μm sections. Sections were immediately fixed in 4% paraformaldehyde for 45 minutes before dehydration and protease incubation. Hybridisation was performed using probes against Ccl8, Ccl24 and Scgb1a1 (Supplementary Table S8), followed by DAPI staining and mounting in ProLong Gold Antifade mountant (Cat# P36930, Thermofisher Scientific, UK). Image capture was carried out using an ImageExpress PICO scanner (Molecular Devices).

***Nanoparticle Tracking Analysis***

CeO_2_NPs were obtained from Sigma Aldrich (Dorset, United Kingdom) (cat # 544841). DEP and CEP particles were suspended in distilled H_2_O (25 µg/mL) before sonication (QSonica Sonicators, CT, USA) with 4.2x105kJ/m3. Particle size distribution was determined using nanoparticle tracking analysis (NanoSight LM10 instrument, NanoSight, Amesbury, UK). Measurements for 3 technical replicates across 3 independent samples were assessed for a minimum of 60 seconds and processed using NTA 3.2 analytical software.

***Endotoxin Assessment***

Endotoxin levels in particles and HDM were assessed using the Pierce Chromogenic Endotoxin Quantification Kit (Cat# A39552, Thermo Scientific, UK). Preparations of 2 mg/mL particles and 1 mg/mL HDM in PBS as equivalent stock solutions used for intranasal instillation procedures were incubated for 24 hrs at 4’C. Solid material was removed by centrifugation (20,000 x g, for 10mins) and the supernatant examined for endotoxin content against known endotoxin kit standards. Results are expressed as EU units per mL.
