# Supplementary material for: Pollutant particles enhance house dust mite induced type 2 inflammation and the recruitment of monocyte derived Cd11c+ Gpnmb+ macrophages to the airway lumen
Source: Part Fibre Toxicol. 2026 Apr 11;23:23. doi: 10.1186/s12989-026-00675-8 (PMC13126997; doi:10.1186/s12989-026-00675-8)

Supplementary Figure S1

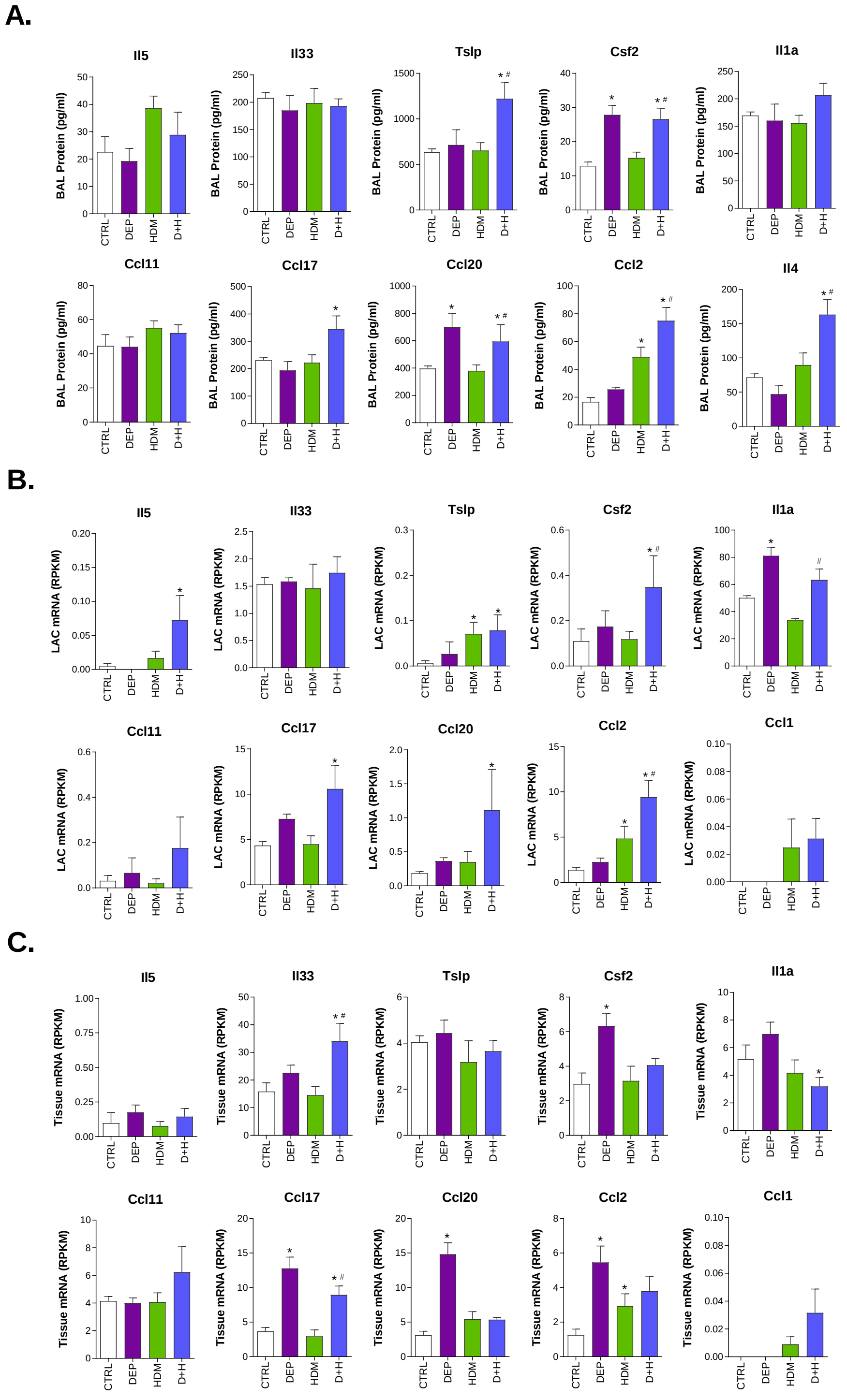

Supplementary Figure S1. *Type 2 inflammatory marker expression within the lung.*

Mice (n=5-7 per group) were treated with HDM and DEP alone or in combination as described in Figure 1A. Luminal airway protein levels for a panel of type 2 inflammatory markers was examined in bronchoalveolar lavage fluid samples and expressed as pg/ml of the original lavage fluid (A). Bulk-seq examination of mRNA levels was also carried out across treatment groups in collected luminal airway cells (LAC) (B) and remaining lung tissue after lavage (Tissue) (C). Results are expressed as normalised reads per kilobase, per million (RPKM). Results are expressed as Mean +/- SEM for each treatment group. Statistical comparisons of treatments are indicated as * (p<0.05) when compared to CTRL levels and as # (p<0.05), when compared to HDM.

Supplementary Figure S2

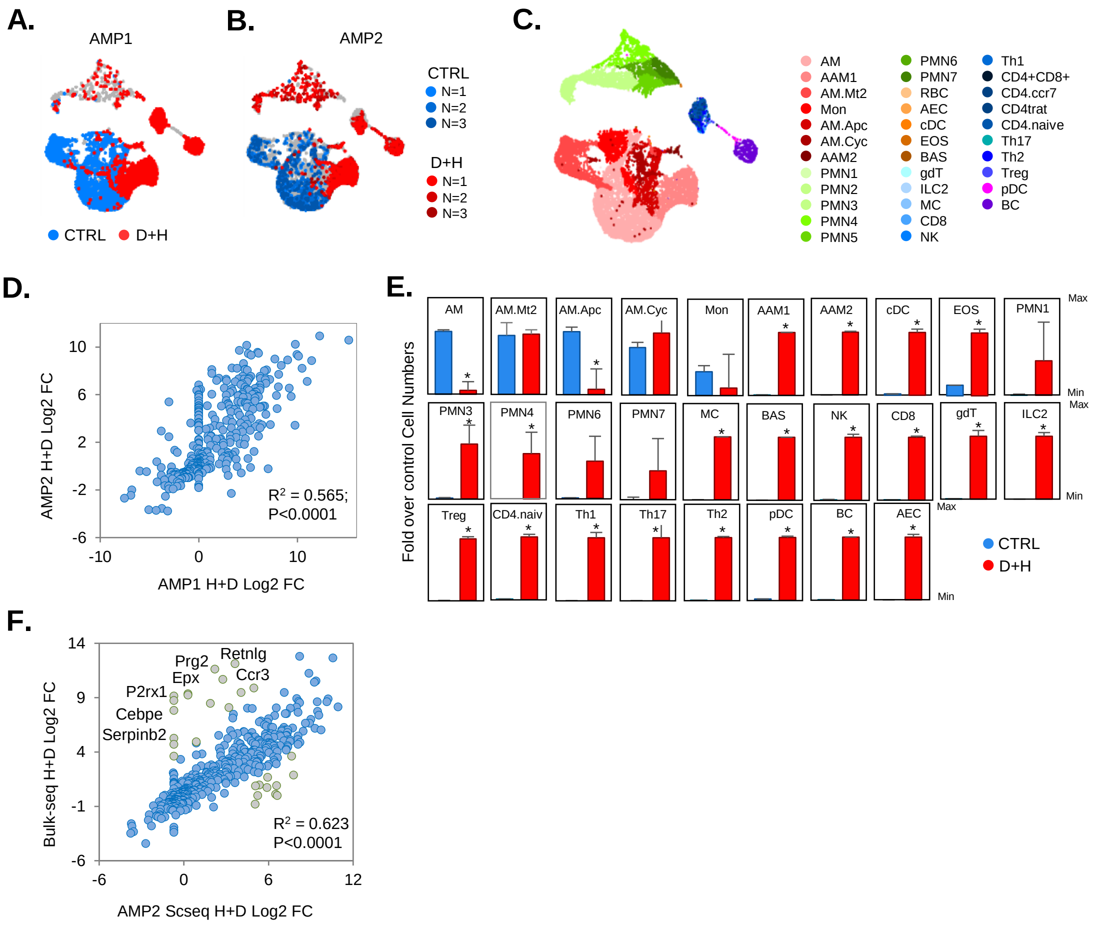

Supplementary Figure S2. *Confirmation of TGE Scseq DEP and HDM effects on luminal airway cell composition and transcriptional specificity using additional datasets.*

A second set of instillation mouse exposures to CTRL and D+H (n=3 per group) was carried out to examine TGE ScSeq and bulkseq changes within the LAC compartment. This new dataset (AMP2) was combined with the original AMP1 data, subjected to UMAP dimensionality reduction, where cells with similar expression patterns clustered together (A-B). Cell types were identified based on unique expression of immune cell specific markers (C). Log 2 fold change (Log2 FC) of D+H over CTRL levels for differentially expressed genes was compared between AMP1 and AMP2 datasets using Pearson corelation and a coefficient of determination (R^2) value and corelation p Value calculated (D). Cell numbers within the combined datasets (AMP1+AMP2) were normalised and comparisons between CTRL and D+H treatments for each cell type was performed (E). Results are expressed as Mean +/- SEM for each treatment group. Statistical comparisons of D+H to CTRL levels is indicated as * ($p<0.05$). Bulkseq was performed on a portion of the same LAC collected for the AMP2 dataset. Log 2 FC changes were calculated and a direct comparison to Log2 FC from the AMP2 Scseq dataset was carried out using Pearson corelation (F). A coefficient of determination (R^2) value and corelation p Value was also calculated. Those genes with the greatest variation between both datasets are highlighted in green (F).

Supplementary Figure S3

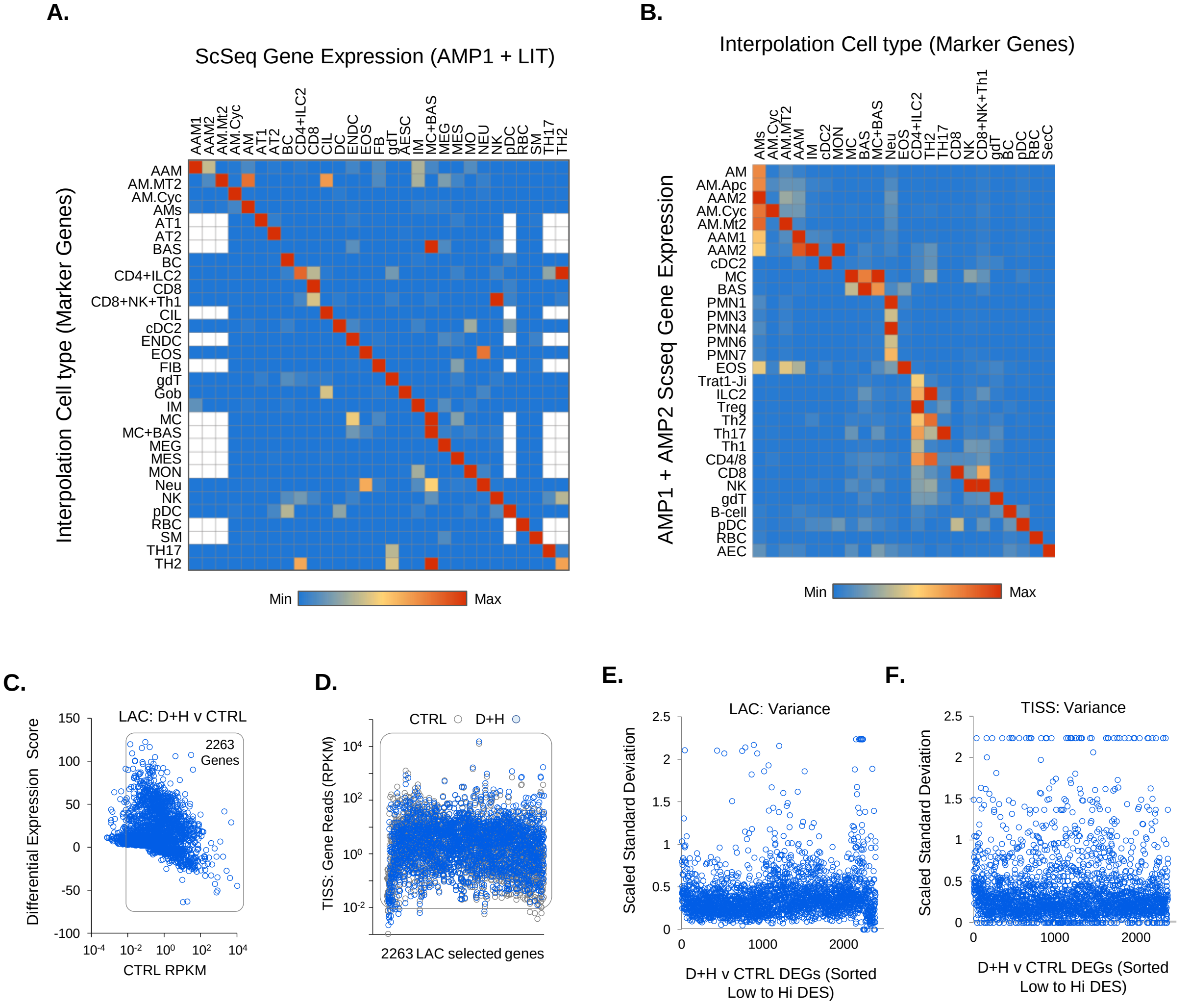

Supplementary Figure S3. *Cell type specificity and sensitivity analysis of transcriptional datasets.*

A comparison between our cell interpolation gene sets (y-axis) to a secondary source of cell type specific gene expression data (x-axis) was carried out to assess specificity of our dataset genes to cell type. The secondary dataset (SD) used a combination of AMP1 data and two additional single cell sequencing datasets (PMID: 33692365, PMID: 37117166). Cell interpolation gene expression data was obtained from the secondary datasets, assigned to SD cell type (x-axis), expression levels normalised to the cell type value with highest expression. Within cell types, normalised gene expression was averaged and plotted against cell interpolation cell type (A). The interpolation gene set was further interrogated for specificity to cell type using a combination of AMP1+AMP2 data (B). LAC D+H differentially expression genes (2 fold change, FDR < 0.05) were selected and RPKM values for CTRL was plotted against the differential expression score (C). Those LAC differentially expressed genes with an RPKM in CTRL >0.01 was identified (C). RPKM data for this subset of genes was extracted from CTRL and D+H TISS data and plotted (D). Standard deviations of fold change values from these D+H v CTRL datasets were scaled (Max 2.25) and plotted individually for LAC (E) and TISS (F) datasets.

Supplementary Figure S4

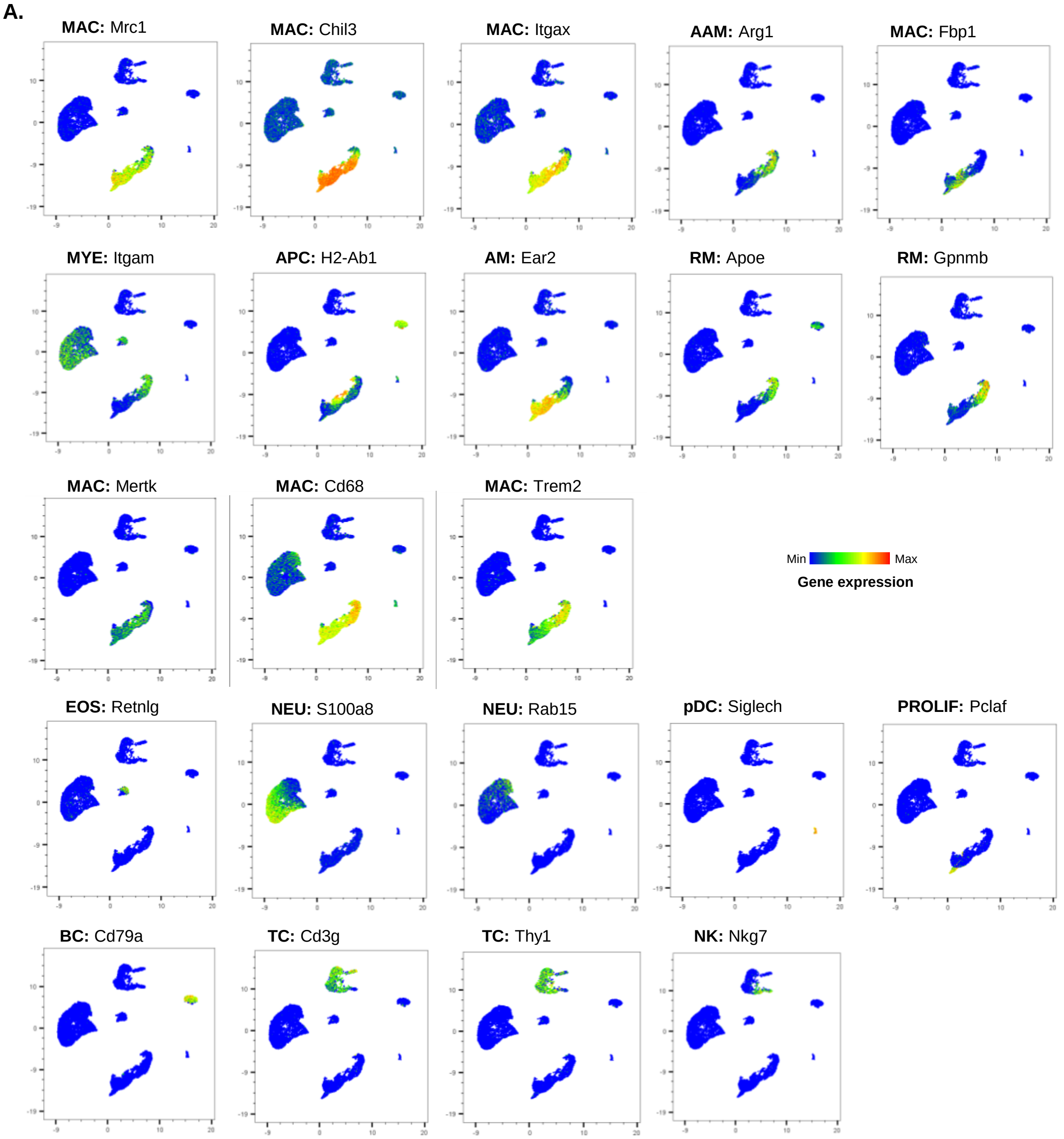

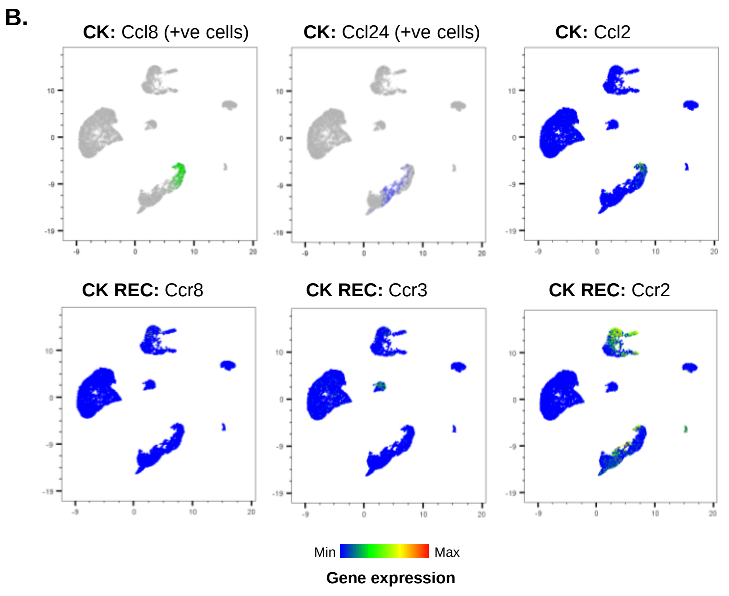

Supplementary Figure S4. *WTA Scseq analysis of luminal airway cell (LAC) populations from HDM and D+H exposed mice.*

WTA Scseq analysis was carried out on LAC isolated from HDM (n=3) and D+H (n=5) repeat exposed mice (9 times over 3 weeks) and cell types (MAC, Macrophage, AAM, Alternatively activated macrophage, NEU; MYE, Myeloid, APC; Antigen presenting cell, AM; Alveolar macrophage, RM; Recruited macrophage, Neutrophil, TC; T-cell, NK; Natural Killer cell, BC; B-cell, EOS; Eosinophil, PROLIF; Proliferating cell, pDC; Plasmacytoid dendritic cell) identified and visualised using UMAP plots (A). Select chemokine (CK) and chemokine receptor (CK REC) expression with LAC are also displayed (B). Expression levels for each gene are normalised to the highest expressing cell, and for Ccl8 and Ccl24 panels, positive cells (>= 1 molecule per cell) are indicated.

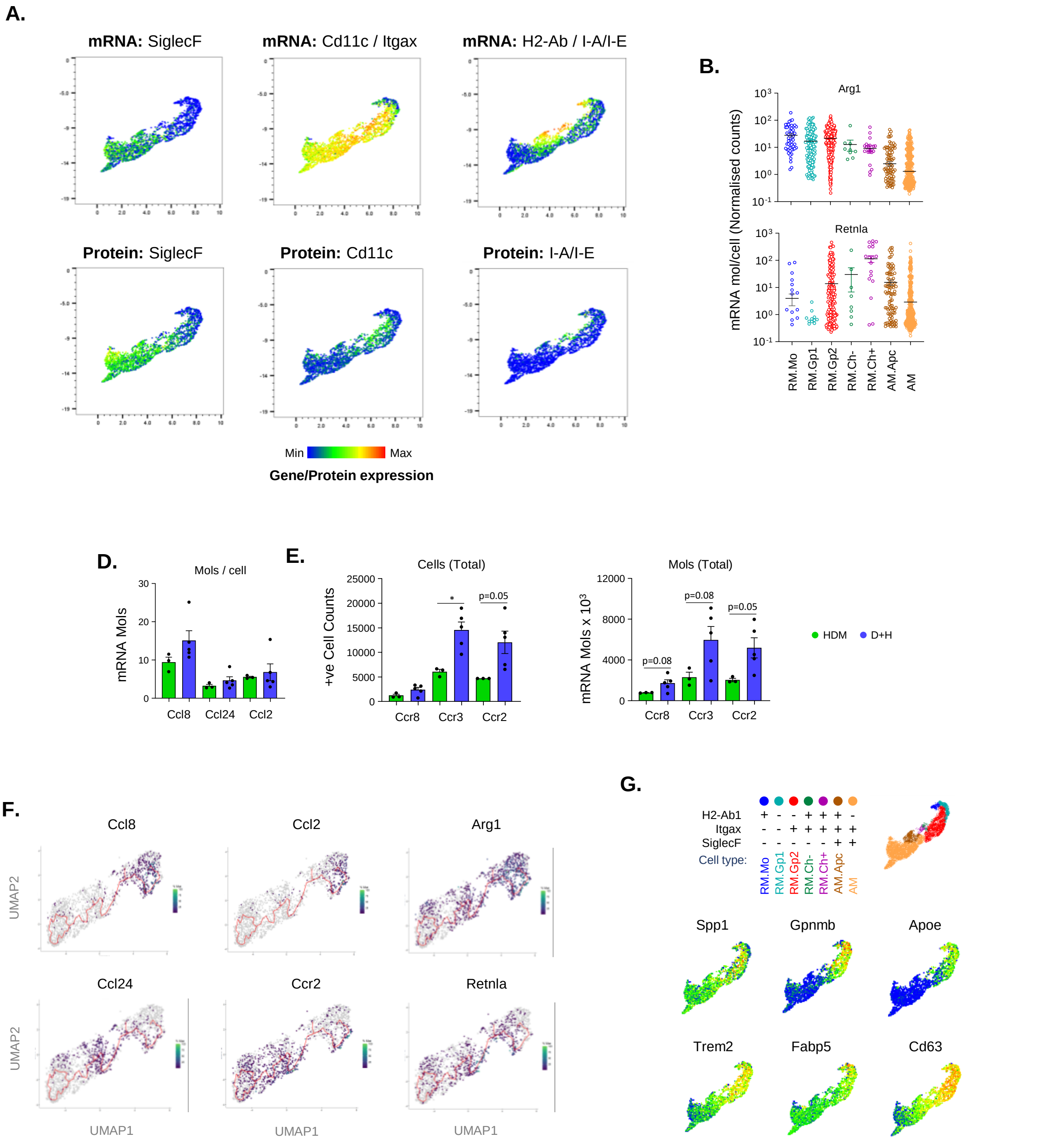

Supplementary Figure S5. *Characterisation of MNP sub-population alterations from WTA Scseq analysis of HDM and D+H luminal airway cells.*

WTA Scseq analysis was carried out on LAC isolated from HDM (n=3) and D+H (n=5) repeat exposed mice (9 times over 3 weeks). MNP cells were examined for expression of classical lung macrophage markers at the mRNA (WTA Scseq) and protein level (Abseq) (A). Expression levels for each marker are normalised to the levels found in the highest expressing cell. mRNA molecule counts per cell was normalised to total cell transcriptome molecule counts and levels for the AAM markers Arg1 and Retnla across MNP sub-populations displayed for each cell (B). The number of molecules per cell across MNP cells was calculated for Ccl8, Ccl24 and Ccl2 (D). Chemokine receptor (Ccr8, Ccr3, Ccr2) expression was examined across total LAC WTA populations (E), with specific mRNA positive cells, adjusted for total LAC content (Left panel) and total mRNA molecules per lung (Right panel) displayed. HDM treatments are indicated as green and D+H as blue. Results are expressed as Mean +/- SEM for each treatment group. Statistical comparisons of D+H v HDM were carried out using unpaired student t-test with significance indicated as * ($p<0.05$) or as specific p values. Expression of select genes mapped onto trajectory analysis UMAP plots are displayed (F).

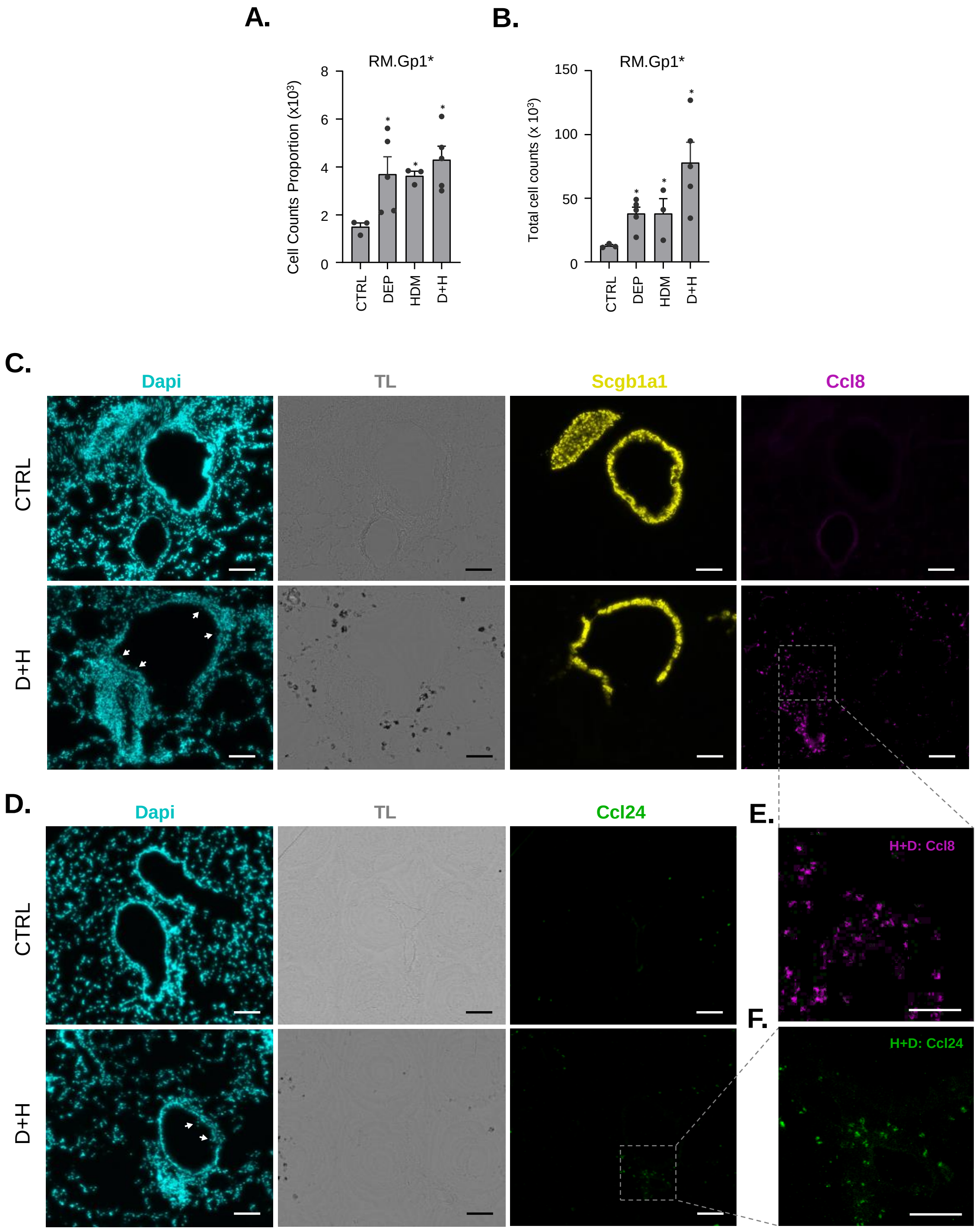

Supplementary Figure S6. *High content imaging of LAC sub-populations and RNAscope analysis of lung tissue.*

Mice were repeatedly exposed through intranasal instillation (9 times over 3 weeks) to CTRL (n=3), DEP (n=5), HDM (n=3) or D+H (n=5). High content imaging for MHCII, Cd11c and SiglecF was used to identify cell types with RM.Gp1* cells identified as negative for all three markers (A-B). Cell counts across treatments is displayed as a proportion of 10,000 analysed cells per mouse (A) or normalised to total LAC cells per lung (B). RNA in situ hybridisation using RNAscope was also carried out on un-lavaged mouse lungs after CTRL and D+H exposures (C-F). In addition to DAPI nuclear stain and transmitted light (TL) imaging for total tissue visualisation, gene specific hybridisation probes for the conducting airway epithelial marker Scgb1a1 and the chemokines Ccl8 and Ccl24 were used to visualise expressing cells within lung tissue sections. White arrows indicate luminal airway cells (LAC) which did not stain for the epithelial marker Scgb1a1. Ccl8 hybridisation is displayed in (C) while Ccl24 is visualised using a separate set of probes (D). Scale bar is 50μm for (C,D) while 25μm for zoomed panels (E,F).

Supplementary Figure S7

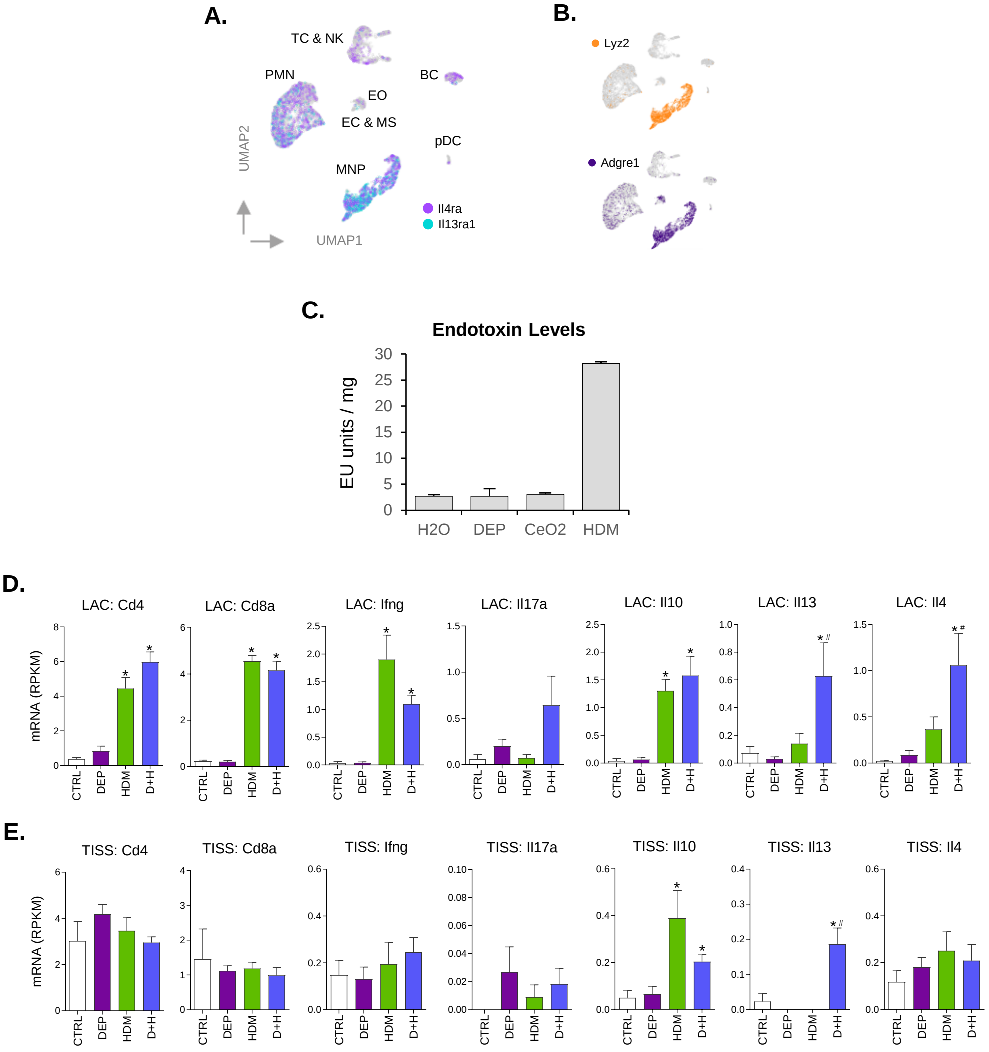

Supplementary Figure S7. *WTA Scseq expression for select genes, endotoxin levels identified in treatment preparations and type 2 mediator mRNA expression in bulk-seq datasets.*

WTA Scseq analysis on LAC isolated from HDM (n=3) and D+H (n=5) repeat exposed mice (9 times over 3 weeks) (A-B). Cell types were identified based on specific marker expression. Cells positive for the type 2 cytokine receptors Il4ra or Il13ra1 (A) or the macrophage markers Lyz2 or Adgre1 (F4/80) (B) are displayed. Endotoxin levels contained within control water, particle and house dust mite preparations used for all exposures were determined using the LAL detection method (C). Results are expressed as EU units per mg of material, or the corresponding volume of water used for stock preparation. (DEP; Diesel exhaust particles, CeO2; Cerium dioxide nanoparticles, HDM; House dust mite extract). Bulk-seq analysis of LAC (D) and TISS (E) levels for T-cell and cytokine markers across exposures alone and in combination are displayed as mRNA normalised counts (RPKM). DEP treatments are indicated as purple, Statistical comparisons for bulk-seq data were carried out using ANOVA with significance indicated as * ($p<0.05$) when compared to CTRL levels and as # ($p<0.05$), when compared to HDM.

Supplementary Figure S8

A.

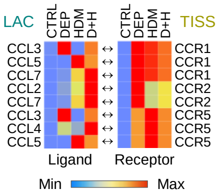

Supplement: Supplementary file 4 — Supplementary Material 4. [file 12989_2026_675_MOESM4_ESM.pdf]
